# Smarce1-dependent modulation of Stat3 signaling governs cardiomyocyte proliferation

**DOI:** 10.1186/s40659-026-00681-2

**Published:** 2026-03-10

**Authors:** Deung-Dae Park, Alena Boos, Antonia Hermann, Sujin Kim, Wolfgang Rottbauer, Steffen Just

**Affiliations:** 1 Molecular Cardiology, Department of Internal Medicine II, University of Ulm, Albert-Einstein-Allee 23, 89081 Ulm, Germany; 2 Department of Internal Medicine II, University of Ulm, Albert-Einstein-Allee 23, 89081 Ulm, Germany

**Keywords:** Cardiomyocyte, Proliferation, Smarce1, SWI/SNF complex, Stat3, Zebrafish

## Abstract

Developmental pathways that govern organ growth during embryogenesis are often reactivated in adult organisms to support tissue regeneration. Despite their profound clinical relevance for cardiac repair, the molecular mechanisms underlying heart growth during development and regeneration remain incompletely understood. Here, we identify Smarce1, a core subunit of the SWI/SNF chromatin-remodeling complex, as a key regulator of cardiomyocyte (CM) proliferation in zebrafish. Using genetically engineered Smarce1 loss-of-function models, we combined heart-specific transcriptomic (RNA-seq, single-cell RNA-seq) and epigenomic (ATAC-seq) profiling to delineate Smarce1-dependent regulatory networks. Smarce1 deficiency enhanced CM proliferative activity by modulating cell cycle dynamics and chromatin accessibility. Integrative analyses revealed that Smarce1 regulates the transcriptional landscape of Stat3 signaling components, thereby linking chromatin remodeling to proliferative signaling. Genetic and pharmacological modulation of Stat3 signaling confirmed its functional contribution to Smarce1-dependent CM proliferation, establishing a conserved Smarce1-Stat3 axis operating during both developmental and adult cardiac growth. Collectively, our findings uncover an epigenetic mechanism by which Smarce1 fine-tunes CM proliferation and identify Smarce1 as a promising target for promoting regenerative CM expansion.

**Supplementary Information:**

The online version contains supplementary material available at 10.1186/s40659-026-00681-2.

## Introduction

Cardiomyocyte (CM) proliferation is fundamental for heart development and indispensable for regenerative responses following injury. In adult mammals, however, most CMs are terminally differentiated and exhibit minimal proliferative capacity, rendering myocardial damage largely irreversible and driving the progression of heart failure [1, 2]. By contrast, zebrafish retain robust regenerative capacity throughout life: pre-existing CMs re-enter the cell cycle and efficiently replace lost myocardium, thereby restoring structure and function [3, 4].

Regenerative and developmental CM proliferation is orchestrated by a finely tuned interplay of transcriptional and epigenetic signaling pathways. Among these, the Signal Transducer and Activator of Transcription 3 (STAT3)—a cytokine-responsive transcription factor—plays a central role in activating gene networks governing proliferation, survival, and tissue regeneration [5]. In zebrafish, Stat3 activation is essential for injury-induced heart regeneration and promotes CM cell-cycle re-entry during myocardial repair [6]. Similarly, in neonatal mice, CM-specific STAT3 deletion compromises regenerative proliferation following cardiac injury [7], and in adult mice recovering from myocarditis, STAT3 activity is required for both CM proliferation and functional recovery [8]. These findings establish STAT3 as a conserved pro-regenerative regulator of CM proliferation across vertebrates. However, the upstream mechanisms controlling STAT3 activation and its integration with chromatin accessibility during cardiac development and regeneration remain insufficiently defined.

Chromatin remodeling represents a key layer of transcriptional control in both development and regeneration. SWItch/Sucrose Non-Fermentable (SWI/SNF) chromatin-remodeling complexes are evolutionarily conserved ATP-dependent remodelers that regulate nucleosome positioning and transcription factor accessibility across tissues [9, 10]. These complexes consist of a catalytic ATPase core and multiple regulatory subunits that confer tissue-specific functions and transcriptional outcomes. SMARCE1, a DNA-binding subunit of the SWI/SNF complex, harbors a high mobility group (HMG) domain that enables recruitment of the complex to promoter and enhancer elements, thereby modulating transcriptional programs [11]. While SWI/SNF components such as Brg1, BAF60c, and BAF250a have been implicated in cardiac morphogenesis and CM proliferation [12–15], the role of Smarce1 in vivo has remained poorly characterized. Our recent work identified Smarce1 as a critical negative regulator of embryonic CM proliferation in zebrafish using an ENU-induced *smarce1*-null mutant, *heart of stone* (*hos*). In *hos* mutants, ventricular wall thickening primarily reflects increased CM number (hyperplasia), and CM-specific Smarce1 overexpression suppresses proliferative activity [16]. However, the upstream chromatin-based mechanisms and signaling pathways linking Smarce1 loss to pro-proliferative transcriptional programs remained unresolved.

Building on these insights, our study delineates the regulatory epigenetic mechanisms through which Smarce1 governs CM proliferation in both the developing and the adult zebrafish heart. Through functional genetic and pharmacological perturbations, combined with heart-specific ATAC-seq, RNA-seq, and single-cell RNA-seq analyses, we identify Smarce1 as an upstream chromatin-based regulator of pro-proliferative Stat3 signaling. Our findings demonstrate that Smarce1 controls DNA accessibility at Stat3-dependent loci, thereby acting as a gatekeeper of CM cell-cycle activity during development.

In conclusion, this work defines Smarce1 as a chromatin-level checkpoint that constrains Stat3 signaling and CM proliferation, providing new mechanistic insight into how epigenetic and transcriptional pathways converge to regulate cardiac growth. These discoveries highlight Smarce1 as a potential therapeutic target to modulate regenerative CM proliferation and advance strategies for cardiac repair and regeneration.

## Results

### Smarce1 deficiency induces cardiomyocyte cell-cycle activity, leading to myocardial hyperplasia

**Fig. 1:**
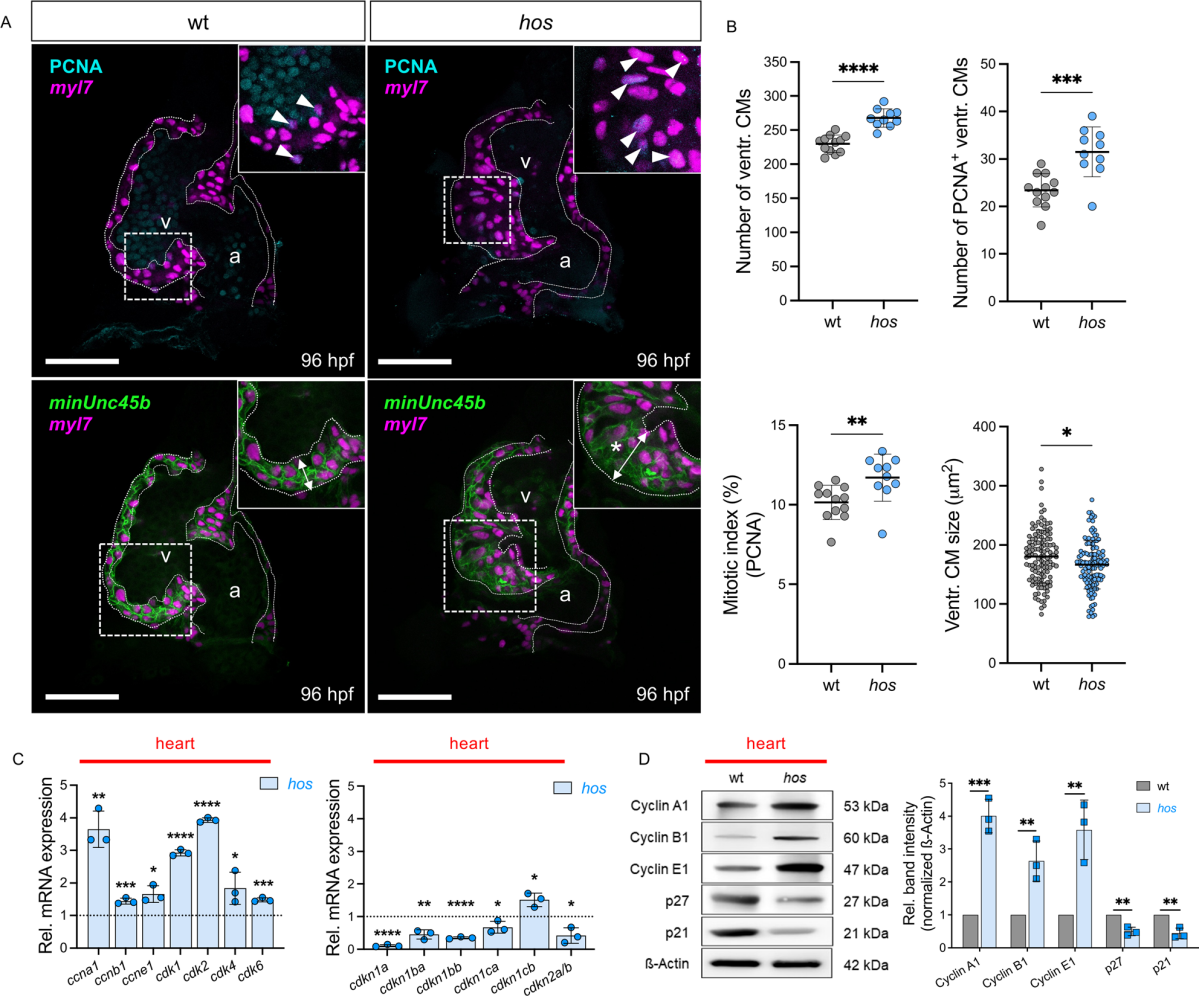
Loss of *smarce1* results in cardiac hyperplasia accompanied by coordinated changes in cell-cycle regulators in the developing heart. (**A**) Confocal projections of fluorescent cardiomyocyte (CM) nuclei (*myl7*.nls) and membrane (*minUnc45b*.CAAX) in embryonic hearts of wt and *heart of stone* (*hos*) at 96 hpf (scale bar: 50 µm). Immunofluorescence (IF) staining of proliferating cell nuclear antigen (PCNA) shows proliferating CMs in wt and *hos* hearts at 96 hpf. The *hos* mutant exhibits a thick ventricular wall (*). White arrows indicate PCNA-positive (PCNA^+^) CMs. (**B**) Quantitative analyses of CM numbers, PCNA^+^ CMs, mitotic index, and size of CMs in wt and *hos* ventricles (Number of ventr. CMs: wt: 229.83 ± 12.80, *hos*: 267.80 ± 13.47, mean difference (*hos - *wt): 37.97, 95% CI: 26.16 to 49.78; Number of PCNA^+^ ventr. CMs: wt: 23.42 ± 3.50, *hos*: 31.50 ± 5.28, mean difference (*hos - *wt): 8.08, 95% CI: 3.92 to 12.24; Mitotic index: wt: 10.15 ± 1.08, *hos*: 11.70 ± 1.47, mean difference (*hos *- wt): 1.55, 95% CI: 0.36 to 2.74, SD, wt: n = 12, *hos*: n = 10 (embryonic hearts); Ventr. CM size: wt: 180.15 ± 45.17, *hos*: 166.68 ± 41.04, mean difference (*hos - *wt): − 13.47, 95% CI: − 24.45 to − 2.49, SD, n = 120 (cells measured across embryos), *p < 0.05, **p < 0.01, ***p < 0.001, ****p < 0.0001). In contrast to wt ventricle, *hos* mutant ventricular wall is thick due to cardiac hyperplasia. (**C**) Quantitative real-time PCR (qRT-PCR) showing relative mRNA expression of cell cycle activators and inhibitors in wt and *hos* hearts at 96 hpf (activators: *ccna1*: 3.65 ± 0.56, *ccnb1*: 1.44 ± 0.09, *ccne1*: 1.66 ± 0.25, *cdk1*: 2.93 ± 0.10, *cdk2*: 3.93 ± 0.07, *cdk4*: 1.84 ± 0.49, *cdk6*: 1.48 ± 0.06; inhibitors: *cdkn1a*: 1.11 ± 0.03, *cdkn1ba*: 0.45 ± 0.14, *cdkn1bb*: 0.38 ± 0.03, *cdkn1ca*: 0.67 ± 0.18, *cdkn1cb*: 1.51 ± 0.21, *cdkn2a/b*: 0.42 ± 0.24, SD, n = 3 (independent biological replicates; pooled-heart mRNA samples), *p < 0.05, **p < 0.01, ***p < 0.001, ****p < 0.0001). (**D**) Heart-specific Western Blot analysis and quantified band intensity of cell cycle proteins in wt and *hos* at 96 hpf (Cyclin A1: 4.00 ± 0.51, Cyclin B1: 2.63 ± 0.61, Cyclin E1: 3.58 ± 0.90, p27: 0.50 ± 0.13, p21: 0.44 ± 0.17, SD, n = 3 (independent biological replicates; pooled-heart lysates), **p < 0.01, ***p < 0.001). v = ventricle, a = atrium, ventr. = ventricular, Rel. = Relative

In a previous study, we identified Smarce1 as a key negative regulator of cardiomyocyte (CM) proliferation in zebrafish using the ENU-induced *heart of stone* (*hos*) mutant, which results in complete loss of Smarce1 expression [16]. Consistent with these findings, Smarce1 deficiency causes abnormal ventricular wall thickening that is driven by increased CM proliferation during cardiac development (Fig. 1A-B). Cell-cycle regulators such as cyclins, cyclin-dependent kinases (CDKs), and cyclin-dependent kinase inhibitors (CDKNs) are well-established master controllers of proliferative activity during development and adulthood across multiple organs, including the heart [17, 18]. Specifically, Cyclin D-CDK2/4/6 and Cyclin E-CDK2 complexes drive the G1-S phase transition, Cyclin A-CDK1/2 controls S phase progression and the G2-M transition, and Cyclin B-CDK1 governs mitosis [19]. Given the cardiac hyperplasia phenotype of *hos*, we next assessed whether Smarce1-deficient hearts exhibit coordinated changes in canonical cell-cycle regulators. In addition to cell-based proliferation readouts, we canonical cyclin/CDK drivers and CDK inhibitors at the RNA and protein levels as molecular corroboration of coordinated cell-cycle regulation. Heart-specific quantitative real-time PCR (qRT-PCR) revealed elevated transcript levels of cell-cycle activators, including *ccna1*, *ccnb1*, *ccnd1*, *ccne1*, *cdk1*, *cdk2*, *cdk4*, and *cdk6*, together with reduced expression of cell-cycle inhibitors such as *cdkn1a/p21*, *cdkn1ba/p27*, *cdkn1bb/p27*, *cdkn1ca/p57*, and *cdkn2a/b/p15* (Fig. 1C). These transcriptional changes were corroborated by heart-specific Western blot assays, which showed increased protein levels of Cyclin A1, Cyclin B1, and Cyclin E1, accompanied by reduced levels of p27 and p21 in *smarce1*-deficient *hos* mutants compared with wild-type siblings (Fig. 1D). Collectively, these findings support that ventricular wall thickening in *hos* mutant hearts results from cardiac hyperplasia associated with excessive CM proliferation and coordinated activation of the canonical cell-cycle regulatory program.

### Smarce1 orchestrates CM proliferation via Jak1/Stat3 signaling

**Fig. 2:**
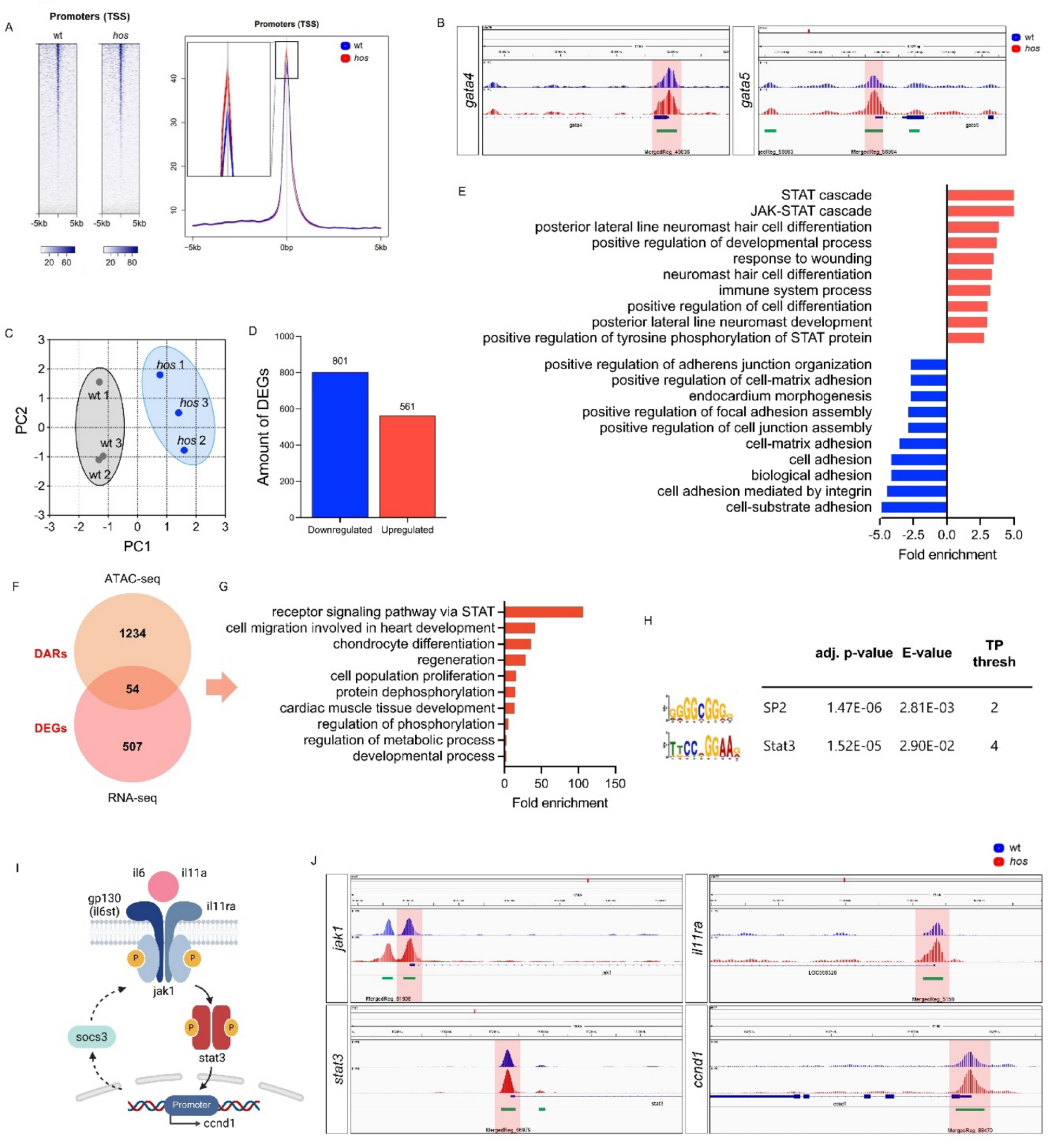
Smarce1 is a key-regulator of Jak/Stat signaling in zebrafish embryonic heart. (**A**) Heatmap visualization and mean signal plot on a promoter (TSS) site of heart-specific ATAC sequencing (ATAC-seq) in wt and *hos* at 96 hpf (ATAC-seq: 60 hearts pooled per genotype). (**B**) Integrative Genomics Viewer (IGV) tracks displaying heart-specific chromatin accessibility at proximal promoter sites (PROX PROM (0–1 kb): red box) of *gata4* and *gata5*. (**C**) Principal component analysis (PCA) plotting the principal component 2 (PC2) of 6 analyzed heart-specific RNA sequencing (RNA-seq) samples against the PC1 (RNA-seq: 3 biological replicates per genotype; total 6 samples). (**D**) Differentially expressed gene (DEG) analysis of heart-specific RNA-seq of wt and *hos* reveals 801 down- and 561 up-regulated genes in *hos* (|log2(fold change: FC)| > 1, p-value < 0.05). (**E**) Gene ontology (GO) term enrichment analysis of upregulated DEGs in *hos* hearts show an induced GO term of *JAK-STAT cascade* (adjusted (adj.) p-value < 0.05). (**F**) Venn diagram showing the overlap between upregulated differentially accessible regions (DARs; merged peaks ≥ 100, log_2_FC ≥ 1, adj. p-value < 0.05) and upregulated DEGs. The light orange circle represents upregulated 1,288 DARs-associated genes unique to ATAC-seq analysis, and the light red circle represents 561 DEGs unique to heart-specific RNA-seq analysis. The overlap contains 54 genes that are both differentially expressed and associated with differentially accessible chromatin regions. (**G**) The subsequent integrative analysis of GO-term enrichment analysis using overlapped 54 genes represents the GO-terms of *receptor signaling pathway *via* STAT3* or *regeneration*. (**H**) Sequence motifs enriched in the genes overlapping between upregulated DARs and DEGs. Motif enrichment analysis identified three highly enriched consensus sequences corresponding to potential SP2- and Stat3-binding motifs, suggesting that the overlapping DEG-DAR gene set is enriched for transcriptional regulators directly relevant to cardiac development and cell proliferation. (**I**) Schematic representation of the Jak1/Stat3 signaling cascade and its transcriptional regulation. (**J**) IGV tracks showing heart-specific chromatin accessibility at proximal promoter sites of *jak1*, *stat3*, *il11ra*, and *ccnd1*

Smarce1 is a functional regulator of the SWI/SNF chromatin remodeling complex, which modulates chromatin accessibility through nucleosome remodeling and thereby controls transcriptional programs. However, the transcriptional targets regulated by Smarce1 in the vertebrate heart remain poorly defined. Previous work demonstrated that Smarce1 binds cis-regulatory regions of the cardiac transcription factor *gata5* to control its expression [20]. To investigate genome-wide chromatin dynamics, we performed heart-specific Assay for Transposase-Accessible Chromatin with high-throughput sequencing (ATAC-seq) in *hos* mutants. Hearts from *hos*^−/−^ × Tg(*myl7*:GFP) embryos and wild-type siblings (*hos*^+/−;+/+^  × Tg(*myl7*:GFP)) were dissected at 96 hpf, subjected to ATAC-seq, and analyzed bioinformatically. We observed globally increased chromatin accessibility in *hos* mutant hearts compared to controls (promoter intervals: wt 44.25%, *hos* 50.82%) (Fig. 2A). Notably, chromatin accessibility was elevated at *gata4* and *gata5* loci (Fig. 2B), supporting earlier evidence for Smarce1-dependent regulation of *gata5* [20].

To assess transcriptional consequences, we performed RNA-seq on dissected 96 hpf *hos* × Tg(*myl7*:GFP) hearts (Fig. 2C). Transcriptomic analysis identified 801 downregulated and 561 upregulated genes in Smarce1-deficient embryonic hearts (Fig. 2D). Strikingly, upregulated genes were enriched for biological processes related to the JAK/STAT cascade, developmental regulation, and cell differentiation (Fig. 2E). Integration of heart-specific RNA-seq and ATAC-seq datasets revealed 54 genes with concomitant increases in expression and chromatin accessibility (Fig. 2F). GO-term analysis of these genes highlighted pathways linked to STAT signaling, regeneration, and cell proliferation (Fig. 2G). Motif enrichment analysis of differentially accessible regions (DARs) identified significant enrichment of Stat3 and SP2 motifs (Fig. 2H). SP2, a transcription factor cooperating with NF-Y, regulates proliferation-associated gene programs and has established roles in neural progenitor proliferation and differentiation [21]. Stat3, a well-characterized driver of CM proliferation and regeneration in zebrafish and neonatal mice [6, 8], was similarly implicated, suggesting that Smarce1 deficiency activates a pro-proliferative transcriptional network. Further analysis of genes within the Jak1/Stat3 signaling pathway (Fig. 2I) revealed increased chromatin accessibility at the promoters of *jak1*, *stat3*, *il11ra*, and the Stat3 target *ccnd1* in *hos* mutant hearts (Fig. 2J).

**Fig. 3:**
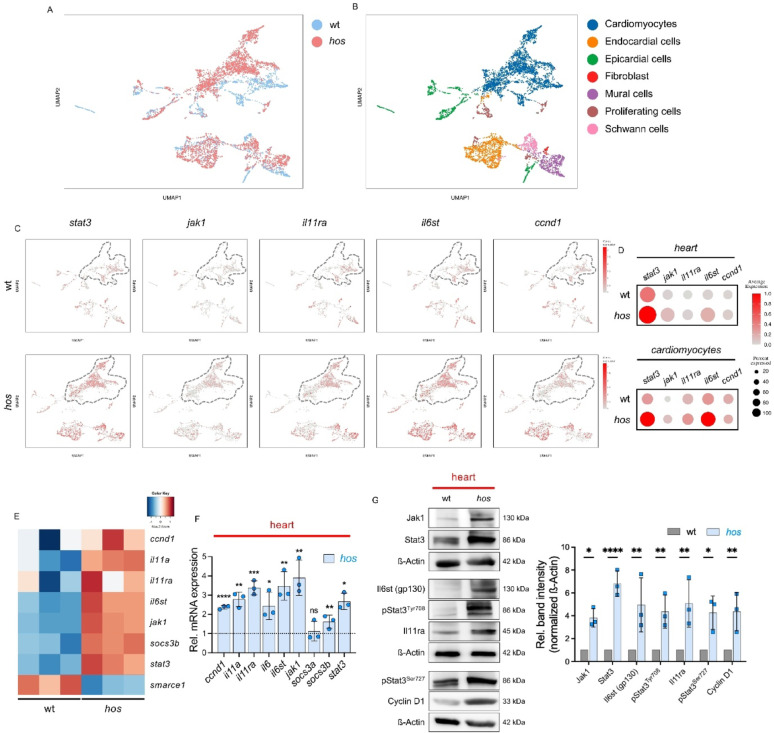
Jak1/Stat3 signaling is upregulated at both transcriptional and translational levels in *hos* heart and cardiomyocytes. (**A**-**B**) Integrated UMAP projections of single-cell RNA-seq data showing cells from dissected hearts of wt and *hos* embryos at 96 hpf. (**A**) UMAP colored by genotype (wt vs. *hos*). (**B**) UMAP depicting distinct cardiac cell-type clusters derived from both genotypes (scRNA-seq: one pooled-heart run per genotype; 2,148 wt cells/4,025 *hos* cells retained after QC). (**C**) Gene expression feature plots illustrating the spatial expression patterns of *stat3*, *jak1*, *il11ra*, *il6st*, and *ccnd1* (colored in red). Color intensity represents relative transcript abundance. Gray dashed lines delineate the cardiomyocyte cluster. (**D**) Dot plots showing the relative expression levels and the proportion of expressing cells for *stat3*, *jak1*, *il11ra*, *il6st*, and *ccnd1* across major cardiac cell populations. Expression of these genes is markedly increased in hearts and cardiomyocytes of *hos* embryos compared to wt. CMs were analyzed at the level of major cardiac cell-type annotations without atrial/ventricular subclustering or cell-cycle phase stratification. (**E**) Visualization of the mean read counts of heart-specific RNA-seq data in wt and *hos* showing Jak1/Stat3 signaling (*ccnd1*, *il11a*, *il11ra*, *il6st*, *jak1*, *socs3b*, and *stat3*) and *smarce1* by heatmap. (**F**) Quantitative RT-PCR results showing heart-specific mRNA expression of Jak1/Stat3 signaling in *hos* compared to wt (*ccnd1*: 2.37 ± 0.10, *il11a*: 2.78 ± 0.38, *il11ra*: 3.38 ± 0.37, *il6*: 2.44 ± 0.71, *il6st*: 3.47 ± 0.74, *jak1*: 3.90 ± 0.91, *socs3a*: 1.12 ± 0.51, *socs3b*: 1.62 ± 0.34, *stat3*: 2.67 ± 0.42, SD, n = 3 (independent biological replicates; pooled-heart mRNA samples), ns: *p* > 0.05, *p < 0.05, **p < 0.01, ***p < 0.001, ****p < 0.0001). (**G**) Western Blot analyses and quantified band intensity displaying the augmented protein expression of Jak1/Stat3 signaling factors in wt and *hos* hearts (Jak1: 3.83 ± 0.81, Stat3: 6.81 ± 1.11, Il6st: 4.96 ± 2.37, pStat3^Tyr708^: 4.36 ± 1.45, Il11ra: 5.08 ± 2.07, pStat3^Ser727^: 4.27 ± 1.47, Cyclin D1: 4.37 ± 1.68, SD, n = 3 (independent biological replicates; pooled-heart lysates), *p < 0.05, **p < 0.01, ****p < 0.0001). pStat3^Tyr708^ denotes the zebrafish Stat3 phosphorylation site recognized by the antibody used in this study. Rel. = Relative

To delineate the cardiac-specific transcriptional consequences of *smarce1* deficiency, we performed single-cell RNA sequencing (scRNA-seq) and comprehensive transcriptomic profiling at single-cell resolution using dissected hearts from wt and *hos* embryos at 96 hpf (Fig. 3A-B). Gene expression analysis of the scRNA-seq data revealed a marked reduction of *smarce1* expression, whereas *gata4* and *gata5* were upregulated in both whole-heart and CM clusters of *hos* embryos (Fig. S1A-B). This pattern was consistent with the increased chromatin accessibility observed at the *gata4* and *gata5* loci in heart-specific ATAC-seq datasets from *hos*.

To elucidate the impact of *smarce1* loss on Jak1/Stat3 signaling, we examined the expression of *stat3*, *jak1*, *il11ra*, *il6st*, and *ccnd1* across all cardiac cell populations. Compared with wt hearts, transcript levels of these genes were elevated in *hos* mutants (Fig. 3C-D). CM-specific transcriptomic profiling further confirmed a pronounced upregulation of Jak/Stat3 pathway components, including *stat3*, *jak1*, *il11ra*, *il6st*, and *ccnd1* (Fig. 3C-D). In addition to CMs, the scRNA-seq dataset also indicates regulation of STAT pathway components, including *stat3*, within endocardial cell populations (Fig. S1C-D). These lineage-resolved expression patterns suggest that Smarce1 deficiency is associated with transcriptional modulation across multiple cardiac cell types. CMs were analyzed at the level of major cell-type annotations, without atrial/ventricular or cell-cycle phase-resolved subclustering. In agreement with the scRNA-seq results, heart-specific bulk RNA-seq revealed increased expression of *ccnd1*, *il11a*, *il11ra*, *il6st*, *jak1*, *socs3b*, and *stat3* (Fig. 3E). Notably, *smarce1* mRNA levels were profoundly reduced, corroborating our genetic, qRT-PCR, and protein data. Heart-specific qRT-PCR independently validated the upregulation of Jak/Stat3 pathway genes (Fig. 3F). Consistently, Western blot analyses of isolated hearts demonstrated elevated protein abundance of Jak1, Stat3, IL6st (gp130), IL11ra, and Cyclin D1, together with increased phosphorylation of Stat3 at Tyr708 and Ser727, indicative of enhanced Stat3 activation in *hos* mutant hearts (Fig. 3G).

Collectively, these findings demonstrate that loss of Smarce1 leads to increased chromatin accessibility and transcriptional activation of the Jak/Stat3 signaling pathway, thereby promoting excessive CM proliferation and cardiac hyperplasia in embryonic zebrafish hearts.

### Stat3 activity drives CM proliferation in the developing zebrafish heart

Based on our integrated epigenetic and transcriptomic data, we next sought to functionally validate the role of Stat3 in CM proliferation during cardiac development using complementary loss- and gain-of-function approaches. To this end, we first injected stat3 morpholino (MO) into wild-type zebrafish embryos and confirmed reduced Stat3 protein abundance, whereas a stat3 mismatch control morpholino did not elicit a comparable reduction (Fig. S2A). Stat3 knockdown induced developmental abnormalities including cardiac edema, reduced body length, and impaired cardiac function at 72 hpf (Fig. S2B-E). Quantification of ventricular CM numbers in Tg(*myl7*:mCherry.nls) embryos injected with KCl, *stat3* mismatch (mm) MO, or *stat3* MO revealed a significant reduction in CM numbers in *stat3* morphants compared to controls (Fig. S2F-G). To assess whether this phenotype was attributable to defective CM proliferation, we performed pH3 immunostaining and EdU incorporation assays. Because pH3 and EdU report distinct cell-cycle stages, we used both assays to independently assess mitotic entry (pH3) and S-phase entry (EdU). Both assays demonstrated a marked reduction in mitotic (pH3⁺) and S-phase (EdU⁺) CMs in *stat3* morphants, indicating impaired M-phase progression and DNA synthesis (Fig. S3). These results establish Stat3 as an essential regulator of embryonic CM proliferation.

**Fig. 4:**
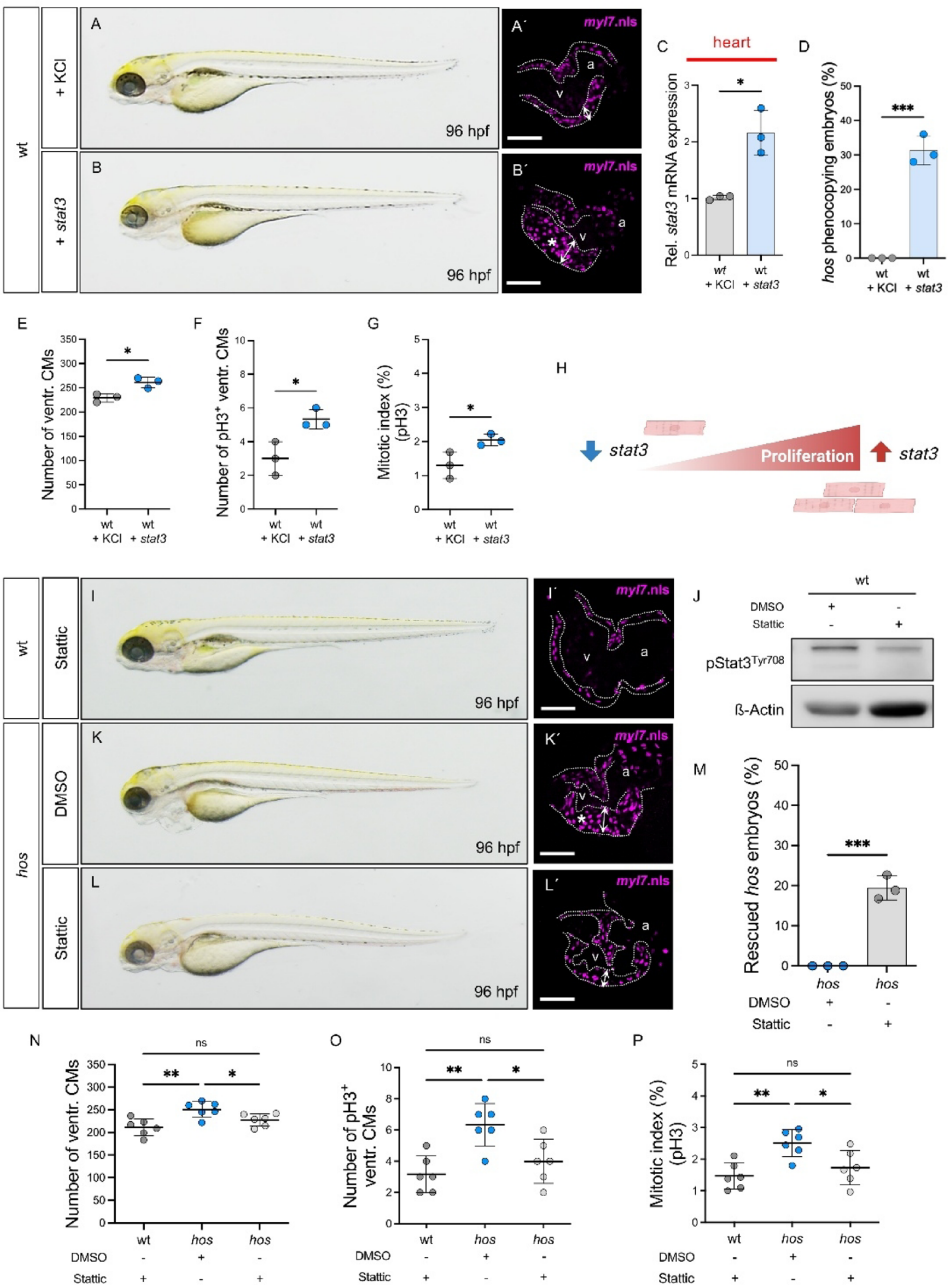
Modification of *stat3* or its activation impacts CM proliferation in the developing heart. (**A**-**B**) Lateral view of wt embryos injected with KCl (control) or wt zebrafish *stat3* mRNA at 96 hpf. (**A´**-**B´**) Confocal images of Tg(*myl7*:mcherry.nls) with KCl or *stat3* mRNA injection at 96 hpf (scale bar: 50 µm). Exogenous *stat3* mRNA injection leads to cardiac edema and a thick ventricular wall (*) compared to the KCl-injected embryonic heart. (**C**) Relative *stat3* expression in the heart of wt embryos with KCl or *stat3* mRNA injection at 96 hpf (wt + KCl: 1.00 ± 0.04, wt + *stat3*: 2.16 ± 0.40, SD, n = 3 (independent biological replicates; pooled-heart mRNA samples), *p < 0.05). (**D**) Quantitative analysis showing the proportion of *stat3* mRNA-injected embryos displaying phenotypic features consistent with the *hos* mutant heart at 96 hpf (wt + *stat3*: 31.33 ± 4.16, n = 50, N = 3 (n = embryos scored; N = independent experiments/clutches), SD, ***p < 0.001). (**E**-**G**) Quantification of ventricular CM numbers, phosphorylated histone 3 positive (pH3^+^) ventricular CMs (pH3 marks mitotic cells), as well as mitotic index in wt embryos injected with KCl or *stat3* mRNA at 96 hpf (Number of ventr. CMs: wt + KCl: 229.33 ± 8.62, wt + *stat3*: 261.00 ± 10.82, mean difference (*stat3 *- KCl): 31.67, 95% CI: 9.05 to 54.29; Number of pH3^+^ ventr. CMs: wt + KCl: 3.00 ± 1.00, wt + *stat3*: 5.33 ± 0.58, mean difference: 2.33, 95% CI: 0.28 to 4.38; Mitotic index: wt + KCl: 1.30 ± 0.39, wt + *stat3*: 2.04 ± 0.17, mean difference: 0.74, 95% CI: − 0.09 to 1.57, SD, n = 3 (embryonic hearts), *p < 0.05). (**H**) Schematic illustrates *stat3* expression levels positively correlate with CM proliferation. (**I**) Lateral view of wt zebrafish embryo treated with low concentration of Stattic (5 µM) at 96 hpf. Stattic was treated from 0 hpf until 96 hpf. (**I´**) A confocal image of dissected heart from low dose of Stattic treated Tg(*myl7*:mcherry.nls) embryo at 96 hpf (scale bar: 50 µm). Low concentration of Stattic doesn’t affect phenotype of whole embryo as well as heart at 96 hpf. (**J**) Western Blot bands displaying protein expression of pStat3^Tyr708^ and ß-Actin in DMSO or Stattic treated embryos at 96 hpf. Stattic reduces Stat3 phosphorylation in zebrafish embryo. (**K**-**L**) Lateral view of *hos* embryos treated with DMSO or low Stattic at 96 hpf. (**K´**-**L´**) Confocal projections of dissected hearts from *hos*(*myl7*:mcherry.nls) embryos after DMSO or low Stattic treatment at 96 hpf. Inhibiting Stat3 activation restored thickened ventricular wall (*) of *hos*. (**M**) Statistical analysis showing the proportion of phenotypically rescued *hos* embryos after Stattic treatment (*hos* + Stattic: 19.40 ± 3.02, n = 50, N = 3 (n = embryos scored; N = independent experiments/clutches), SD, ***p < 0.001). (**N**-**P**) Quantitative analyses of ventricular CM numbers, ventricular pH3^+^ CMs, and mitotic index in the developing zebrafish hearts of wt or *hos* after treatment of DMSO or Stattic (Number of ventr. CMs: wt + Stattic: 211.33 ± 18.91, *hos* + DMSO: 250.83 ± 17.12, *hos* + Stattic: 227.50 ± 13.55, mean difference (*hos* + Stattic - *hos* + DMSO): − 23.33, 95% CI: − 43.33 to − 3.33; Number of pH3^+^ ventr. CMs: wt + Stattic: 3.17 ± 1.17, *hos* + DMSO: 6.33 ± 1.37, *hos* + Stattic: 4.00 ± 1.41, mean difference: − 2.33, 95% CI: − 4.12 to − 0.54; Mitotic index: wt + Stattic: 1.47 ± 0.42, *hos* + DMSO: 2.50 ± 0.43, *hos* + Stattic: 1.74 ± 0.54, mean difference: − 0.76, 95% CI: − 1.39 to − 0.13, SD, n = 6 (embryonic hearts), ns: *p* > 0.05, *p < 0.05, **p < 0.01). Stattic treatment attenuates a hyperplastic heart of *hos* mutants. v = ventricle, a = atrium, ventr. = ventricular, Rel. = Relative

Conversely, overexpression of *stat3* by mRNA injection at the one-cell stage induced a *hos*-like phenotype at 96 hpf, characterized by cardiac edema and ventricular wall thickening (Fig. 4A-D). Quantitative analyses and pH3 immunostaining revealed a significant increase in ventricular CM numbers and proliferative activity in *stat3*-overexpressing embryos (Fig. 4E-G), phenocopying the hyperproliferative phenotype of *hos* mutants. Collectively, these findings demonstrate that Stat3 dosage critically regulates CM proliferative capacity, with loss of Stat3 impairing and gain of Stat3 enhancing CM proliferation during zebrafish heart development (Fig. 4H).

### Pharmacological Stat3 inhibition rescues cardiac hyperplasia in *hos*

To further interrogate the regulatory function of Smarce1 in CM proliferation via Stat3 signaling, we pharmacologically inhibited Stat3 activity in *hos* embryos using the Stat3 phosphorylation inhibitor Stattic [22]. We first optimized Stattic treatment by identifying a concentration that efficiently suppressed pStat3 expression while avoiding developmental abnormalities. Continuous exposure to Stattic from 0 to 96 hpf markedly reduced pStat3 levels at 96 hpf without inducing morphological or physiological defects (Fig. 4I-J). We then applied a subthreshold concentration of Stattic to *hos* embryos to assess whether Stat3 inhibition could ameliorate the hyperplastic phenotype. Remarkably, Stattic treatment alleviated the ventricular wall thickening in *hos* embryos, resulting in a significantly rescued phenotype of 19.40 ± 3.02% at 96 hpf (Fig. 4K-M). Quantitative analysis of ventricular CM numbers together with pH3 immunostaining further demonstrated that pharmacological Stat3 inhibition attenuated the excessive CM proliferation characteristic of the *hos* mutant heart (Fig. 4N-P). Collectively, these findings provide functional evidence that Smarce1 restrains CM proliferation through modulation of Stat3 signaling during zebrafish heart development.

### Myocardium-specific s*tat3* overexpression promotes CM proliferation in the zebrafish heart

**Fig. 5:**
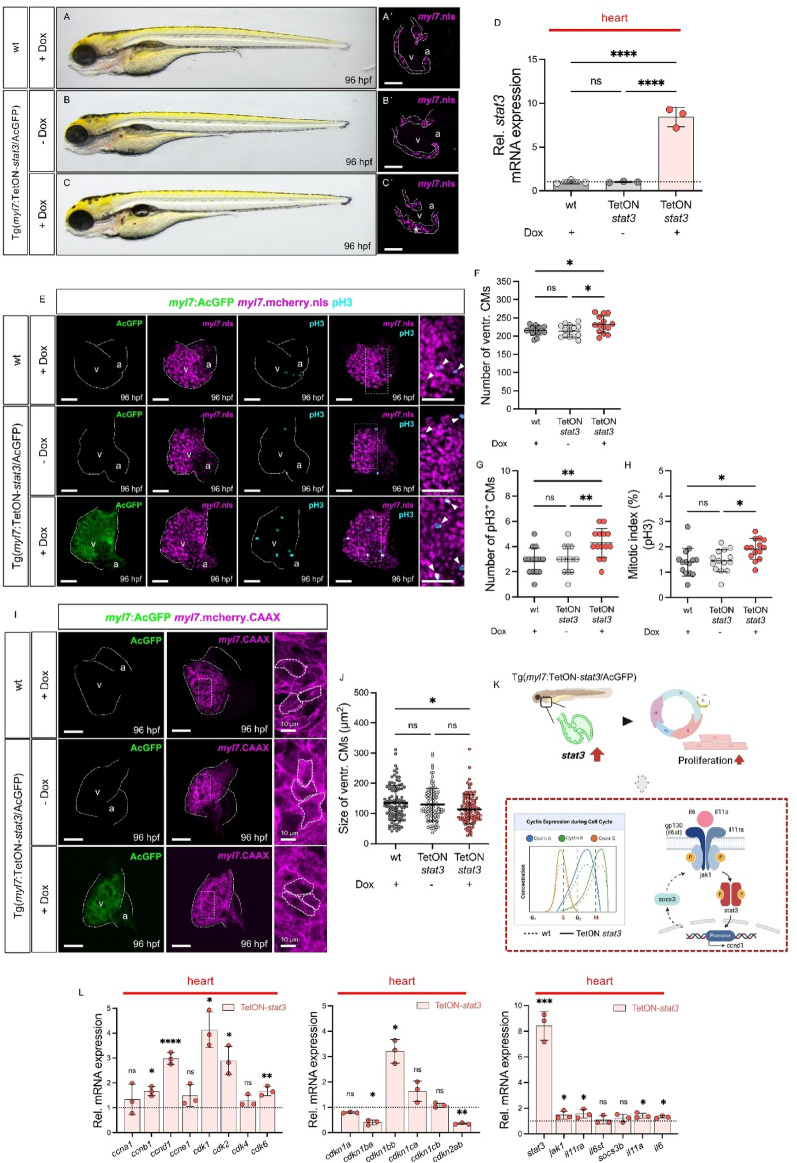
Myocardium-specific overexpression of *stat3* leads to cardiac hyperplasia by upregulating CM proliferation in the developing heart. (**A**-**C**) Lateral view of wt and Tg(*myl7*:TetON-*stat3*/AcGFP) zebrafish embryo with or without doxycycline (dox) treatment at 96 hpf. (**A´**-**C´**) Confocal images of dissected hearts from Tg(*myl7*:mcherry.nls) and Tg(*myl7*:TetON-*stat3*/AcGFP) × Tg(*myl7*:mcherry.nls) embryos with or without dox treatment at 96 hpf (scale bar: 50 µm). Dox-treated TetON-*stat3* embryo exhibited a thick ventricular wall (*). (**D**) Transcriptional level of *stat3* in the hearts of wt or Tg(*myl7*:TetON-*stat3*/AcGFP) embryos showing significant *stat3* overexpression by dox treatment at 96 hpf (wt + dox: 1.00 ± 0.21, TetON-*stat3* − dox: 1.01 ± 0.07, TetON-stat3 + dox: 8.42 ± 1.11, SD, n = 3 (independent biological replicates; pooled-heart mRNA samples), ns: *p* > 0.05, ****p < 0.0001). (**E**) Confocal images of the dissected embryonic hearts from Tg(*myl7*:mcherry.nls) and Tg(*myl7*:TetON-*stat3*/AcGFP) × Tg(*myl7*:mcherry.nls) stained with pH3 at 96 hpf (scale bar: 50 µm). White arrows indicate pH3^+^ CMs. (**F**-**H**) Quantitative analyses of ventricular CM numbers, pH3+ ventricular CMs, and mitotic index (Number of ventr. CMs: wt + dox: 215.00 ± 12.73, TetON-*stat3* − dox: 214.21 ± 16.58, TetON-*stat3* + dox: 231.71 ± 23.20, mean difference (TetON-*stat3* + dox - TetON-*stat3* − dox): 17.50, 95% CI: 1.75 to 33.25; Number of pH3^+^ CMs: wt + dox: 2.86 ± 1.03, TetON-*stat3* − dox: 3.00 ± 1.04, TetON-*stat3* + dox: 4.29 ± 1.14, mean difference: 1.29, 95% CI: 0.44 to 2.14; Mitotic index: wt + dox: 1.40 ± 0.55, TetON-*stat3* − dox: 1.45 ± 0.43, TetON-*stat3* + dox: 1.91 ± 0.41, mean difference: 0.46, 95% CI: 0.13 to 0.79, SD, n = 14 (embryonic hearts), ns: *p* > 0.05, *p < 0.05, **p < 0.01). (**I**) Confocal images of embryonic hearts dissected from wt and Tg(*myl7*:TetON-stat3/AcGFP) crossed with Tg(*myl7*:mcherrry_CAAX), showing fluorescently labeled CM membrane (*myl7*.CAAX) at 96 hpf (scale bar: 50 µm). (**J**) Quantitative analysis of CM size in wt and TetON-*stat3* ventricles at 96 hpf (wt + dox: 153.13 ± 58.55, TetON-*stat3 *− dox: 128.85 ± 54.57, TetON-*stat3* + dox: 113.50 ± 48.44, mean difference (TetON-*stat3* + dox - TetON-*stat3* − dox): − 15.35, 95% CI: − 29.74 to − 0.96, SD, n = 100 (cells measured across embryos), ns: *p* > 0.05, *p < 0.01). (**K**) Schematic overview of enhanced CM proliferation in the zebrafish embryonic heart by conditional *stat3* overexpression, which could contribute to the activation of cell cycle and Jak1/Stat3 signaling. (**L**) Quantitative RT-PCR results showing heart-specific mRNA expression of cell cycle factors and Jak1/Stat3 signaling in TetON-*stat3* compared to wt after dox treatment (cell cycle activators: *ccna1*: 1.34 ± 0.62, *ccnb1*: 1.66 ± 0.19, *ccnd1*: 2.98 ± 0.24, *ccne1*: 1.48 ± 0.44, *cdk1*: 4.13 ± 0.72, *cdk2*: 2.90 ± 0.57, *cdk4*: 1.28 ± 0.24, *cdk6*: 1.67 ± 0.19; cell cycle inhibitors: *cdkn1a*: 0.79 ± 0.02, *cdkn1ba*: 0.40 ± 0.10, *cdkn1bb*: 3.21 ± 0.46, *cdkn1ca*: 1.64 ± 0.41, *cdkn1cb*: 1.08 ± 0.09, *cdkn2a/b*: 0.35 ± 0.04; Jak1/Stat3 signaling: *stat3*: 8.42 ± 1.11, *jak1*: 1.47 ± 0.31, *il11ra*: 1.57 ± 0.36, *il6st*: 1.09 ± 0.32, *socs3b*: 1.23 ± 0.31, *il11a*: 1.42 ± 0.21, *il6*: 1.31 ± 0.10, SD, n = 3 (independent biological replicates; pooled-heart mRNA samples), ns: p > 0.05, *p < 0.05, **p < 0.01, ***p < 0.001, ****p < 0.0001). v = ventricle, a = atrium, ventr. = ventricular, Rel. = Relative

Stat3 signaling is a well-established regulator of cell-cycle activity across diverse cell types [5], including CMs during zebrafish heart regeneration [6]. This function is mediated, at least in part, through the transcriptional activation of Cyclin D1 [23]. However, the impact of myocardium-specific *stat3* overexpression on CM proliferation during heart development has not yet been defined. To address this, we generated an inducible, CM-specific *stat3* overexpression model, Tg(*myl7*:TetON-*stat3*/AcGFP), enabling myocardium-restricted *stat3* induction upon doxycycline administration (Fig. S4). Doxycycline treatment robustly induced *stat3* expression in embryonic hearts at 96 hpf, whereas wild-type embryos treated with doxycycline or Tg(*myl7*:TetON-*stat3*/AcGFP) embryos without induction showed baseline *stat3* levels (Fig. 5D). While no gross embryonic morphological abnormalities were detected (Fig. 5A-C), ventricular walls were significantly thickened in doxycycline-treated Tg(*myl7*:TetON-*stat3*/AcGFP) embryos compared to controls (Fig. 5A´-C´).

To determine whether this phenotype reflected increased CM proliferation, we performed pH3 immunostaining and EdU incorporation assays on Tg(*myl7*:TetON-*stat3*/AcGFP × Tg(*myl7*:mCherry.nls) embryos at 96 hpf (Fig. 5E, S5A). Quantitative analysis revealed significantly elevated ventricular CM numbers driven by enhanced CM proliferation in Stat3-induced hearts (Fig. 5F-H, S5B-D), closely resembling the hyperplastic phenotype observed in *hos* mutants. To distinguish between hyperplasia and hypertrophy, we crossed Tg(*myl7*:TetON-*stat3*/AcGFP) with Tg(*myl7*:mCherry.CAAX) and assessed ventricular CM size following doxycycline induction (Fig. 5I). CM size remained unchanged, confirming that Stat3 overexpression promotes myocardial hyperplasia rather than hypertrophy (Fig. 5J). Finally, heart-specific qRT-PCR analysis of doxycycline-induced Tg(*myl7*:TetON-*stat3*/AcGFP) embryos demonstrated activation of CM cell-cycle regulators and key Jak1/Stat3 signaling components, mirroring transcriptional changes observed in Smarce1-deficient *hos* hearts (Fig. 5K-L).

Together, these findings establish that myocardium-specific Stat3 overexpression is sufficient to activate Jak1/Stat3 signaling, drive CM cell-cycle re-entry, and promote myocardial hyperplasia during zebrafish heart development.

### Smarce1 and Stat3 are key regulators of CM proliferation in the adult zebrafish heart

**Fig. 6:**
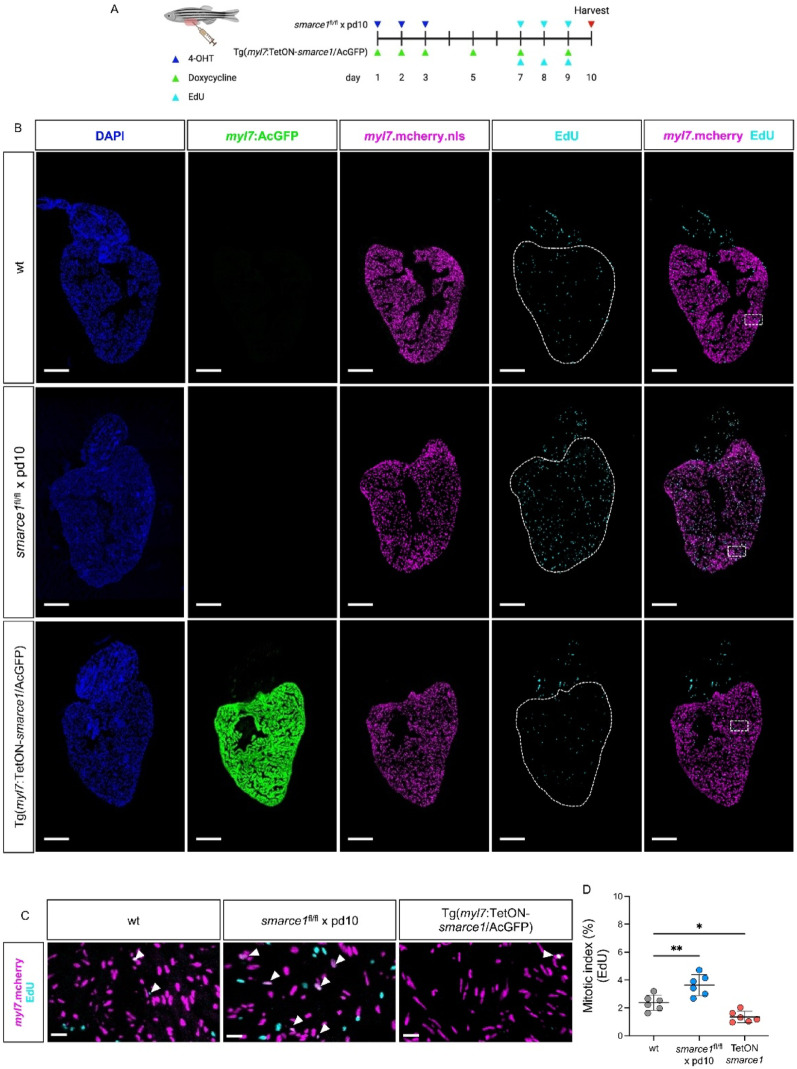
Smarce1 regulates proliferative potential of CMs in the adult zebrafish heart. (**A**) Scheme of experimental plan to modulate *smarce1* expression in the adult zebrafish myocardium by using models of *smarce1*^fl/fl^ × Tg(*cryaa*:DsRed,-5.1*myl7*:CreERT2)^pd10^ or Tg(*myl7*:TetON-*smarce1*/AcGFP). (**B**) Confocal microscopy images of EdU-incorporated (EdU labels S-phase entry) adult zebrafish hearts from wt, *smarce1*^fl/fl^ × pd10, and Tg(*myl7*:TetON-*smarce1*/AcGFP) crossed with Tg(*myl7*:mcherry.nls) (scale bar: 200 µm). (**C**) Magnified IF images of EdU-incorporated adult zebrafish hearts of (B) (white arrow: EdU^+^ CM; scale bar: 20 µm). (**D**) Statistical analysis of mitotic index in wt and *smarce1*-modulated adult zebrafish hearts (wt: 2.37 ± 0.56*, smarce1*^*fl/fl*^ × pd10: 3.64 ± 0.76, TetON-*smarce1*: 1.35 ± 0.40, mean difference (*smarce1*^*fl/fl*^ × pd10 - wt): 1.27, 95% CI: 0.40 to 2.14, mean difference (TetON-*smarce1 - *wt): − 1.02, 95% CI: − 1.66 to − 0.38, SD, n = 6 (adult hearts), *p < 0.05, **p < 0.01)

Proliferation of CMs in the adult zebrafish heart is robustly stimulated by injury, enabling complete regeneration of the damaged myocardium. To explore whether CM proliferation can also be modulated in the uninjured adult heart through manipulation of *smarce1*, we employed both a CRISPR/Cas9-based Cre/loxP knockout strategy and an inducible TetON system. For conditional CM-specific inactivation of *smarce1*, we generated *smarce1*^*fl/fl*^ zebrafish and crossed them with the Cre-driver line Tg(*cryaa*:DsRed,-5.*1myl7*:CreERT2)^pd10^, in combination with Tg(*myl7*:mCherry.nls) to visualize CM nuclei (Fig. 6A). EdU incorporation assays revealed that, following 4-hydroxytamoxifen (4-OHT) administration, *smarce1*^*fl/fl*^ × pd10 adults displayed a significant increase in EdU⁺ CMs within the ventricular myocardium (Fig. 6B-D), indicating that loss of Smarce1 is sufficient to activate CM proliferation under physiological conditions. Conversely, CM-specific overexpression of *smarce1* using the transgenic line Tg(*myl7*:TetON-*smarce1*/AcGFP) [16] and doxycycline induction resulted in a reduced proportion of EdU⁺ CMs in the adult ventricle compared to controls (Fig. 6B-D), suggesting that elevated Smarce1 suppresses CM proliferative activity.

**Fig. 7:**
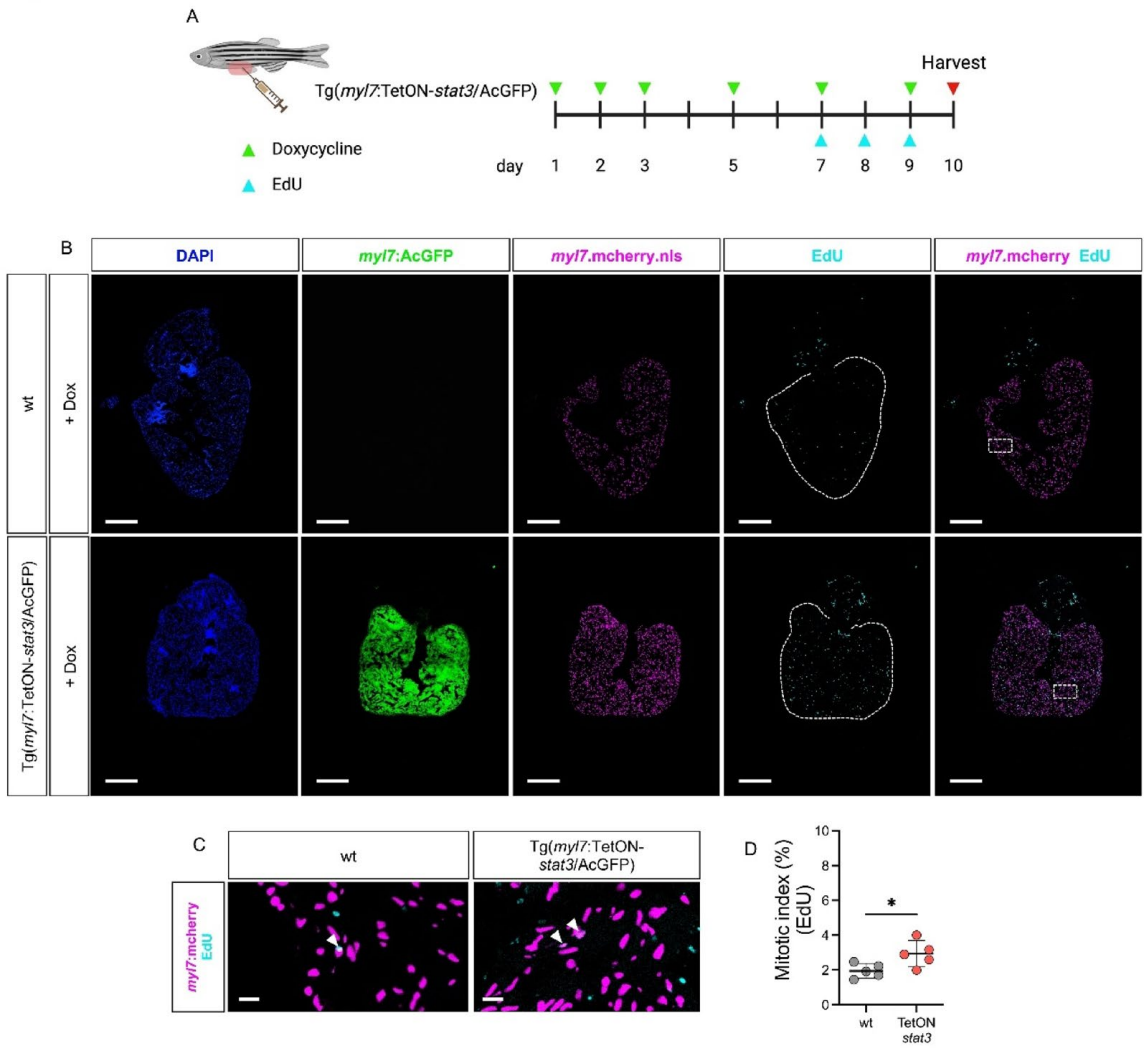
Overexpression of *stat3* potentiates CM proliferation in the adult zebrafish heart. (**A**) Scheme of experimental plan for inducing myocardial *stat3* overexpression in the adult zebrafish heart by using Tg(*myl7*:TetON-*stat3*/AcGFP). (**B**) Confocal images of EdU incorporation assay in the hearts of Tg(*myl7*:mcherry.nls) and Tg(*myl7*:TetON-*stat3*/AcGFP) × Tg(*myl7*:mcherry.nls) with dox-treatment (scale bar: 200 µm). (**C**) Enlarged confocal images of EdU-incorporated CMs in the hearts of wt and Tg(*myl7*:TetON-*stat3*/AcGFP) crossed with Tg(*myl7*:mcherry.nls) (white arrow: EdU^+^ CM, scale bar: 20 µm). (**D**) Statistical analysis of mitotic index in the hearts of wt and Tg(*myl7*:TetON-*stat3*/AcGFP) treated with dox (wt: 1.96 ± 0.41, TetON-*stat3*: 2.94 ± 0.74, mean difference (TetON-*stat3 - *wt): 0.98, 95% CI: 0.06 to 1.90, SD, n = 5 (adult hearts), *p < 0.05). Dox = doxycycline

To further assess the role of Stat3 in the adult myocardium, we induced *stat3* overexpression in Tg(*myl7*:TetON-*stat3*/AcGFP × Tg(*myl7*:mCherry.nls) adults via doxycycline treatment combined with EdU administration (Fig. 7A). Similar to the effects observed in *smarce1*^*fl/fl*^ × pd10 zebrafish, myocardium-specific induction of *stat3* led to a significant increase in EdU⁺ CMs compared to wild-type controls (Fig. 7B-D). These findings demonstrate that Stat3 upregulation is sufficient to enhance CM proliferative potential in the adult zebrafish heart.

Collectively, our results establish that Smarce1 governs CM proliferation in both the embryonic and adult zebrafish heart through epigenetic modulation of the Jak1/Stat3 signaling axis.

## Discussion

In this study, we combine omics-based and genetic approaches to demonstrate that loss of Smarce1 stimulates Stat3 signaling, thereby unleashing a pro-proliferative CM program. The ability of *stat3* overexpression to phenocopy the *heart of stone* (*hos*) mutant and the rescue of *hos* cardiac hyperplasia by pharmacological Stat3 inhibition underscore the causal role of the Smarce1-Stat3 axis across both developmental and adult stages in zebrafish. Because Stattic is not perfectly specific, we interpret the pharmacological inhibition experiments as supportive evidence and emphasize the convergence with independent genetic perturbations of Stat3 signaling. The convergence of cardiac-specific ATAC-seq, RNA-seq and single-cell RNA-seq findings on Stat3-regulated loci further establishes Smarce1 as an upstream epigenetic regulator of Stat3-dependent transcriptional networks. A limitation of our study is that we did not directly assess Smarce1-dependent Stat3 occupancy at individual target loci within CMs. Thus, our model is based on convergent accessibility and transcriptional signatures together with functional perturbation data. Future CM-enriched CUT&RUN/CUT&Tag or ChIP-based profiling will be important to define locus-specific Stat3 binding and its dependence on Smarce1. Importantly, our data place Smarce1 at the intersection of chromatin remodeling and cytokine signaling, suggesting that epigenetic control of Stat3 accessibility is a decisive mechanism for modulating CM proliferative potential. Compared with bulk RNA-seq, single-cell RNA-seq enabled cell-type resolution that allowed us to attribute the Smarce1-dependent transcriptional changes to specific cardiac lineages, most prominently in CMs, rather than averaging signals across the entire heart. In addition, the single-cell dataset captured regulatory changes in non-CM populations that may be diluted in bulk measurements, thereby complementing our bulk RNA-seq and ATAC-seq analyses with a lineage-resolved context.

Consistent with this chromatin-signaling interface, STAT3 is increasingly appreciated beyond the heart as a chromatin-coupled transcription factor whose transcriptional output can be modulated by epigenetic regulators in developmental and oncogenic contexts. For example, p300/CBP-dependent acetylation of STAT3 (Lys685) has been reported to stabilize STAT3 dimerization and enhance transcriptional activity, linking cytokine signaling to chromatin-associated coactivator function [24]. In addition, Polycomb-associated mechanisms have been implicated in reinforcing STAT3-driven proliferative programs; EZH2 phosphorylation has been shown to promote STAT3 methylation and STAT3 signaling in glioblastoma stem-like cells [25]. In CMs, proliferative signaling operates under strong structural and cell-cycle control constraints, and we therefore propose that Smarce1/SWI/SNF-dependent modulation of chromatin accessibility may serve as a context-specific mechanism that shapes Stat3 target engagement and helps limit excessive hyperplastic growth.

Through cardiac-specific ATAC-seq, RNA-seq and single-cell RNA-seq, we identified increased chromatin accessibility and concomitant transcriptional activation of pathways associated with cell differentiation, developmental processes, and Jak/Stat3 signaling in *hos* mutant. Functional validation revealed that Stat3 activation is sufficient to trigger CM proliferation in both embryonic and adult zebrafish, supporting the essential role of Jak1/Stat3 signaling in cardiac growth. This aligns with prior studies demonstrating that Stat3 orchestrates proliferation in diverse contexts: mediating G-CSF-induced proliferation of mouse embryonic stem cell-derived CMs [26], driving intrauterine G-CSF-dependent cardiac hyperplasia, and regulating G1-S and G2-M transitions by modulating cyclins and CDKs [27, 28]. In line with these mechanisms, we observed upregulation of *ccnb1*, *ccnd1*, *cdk1*, *cdk2*, and *cdk6* alongside suppression of cell cycle inhibitors in Smarce1-deficient and Stat3-overexpressing embryonic zebrafish hearts. Taken together, these results establish Smarce1-mediated Jak1/Stat3 signaling as a critical regulator of cell cycle progression and CM proliferation during zebrafish heart development.

While our transcriptomic and functional analyses confirm that excessive CM proliferation underlies ventricular wall thickening in *hos* and *stat3*-overexpressing embryos, contributions from non-CM populations cannot be fully excluded. Consistent with this possibility, our scRNA-seq dataset also indicates regulation of STAT pathway components, including *stat3*, in endocardial cell populations, although we did not functionally interrogate endocardial STAT pathway activity in the present study. In our prior analysis, ventricular endocardial cell number was not significantly altered in *hos* at 96 hpf, based on quantification of *fli1*:EGFP-positive endocardial cells [16]. Lineage-tracing has shown that epicardial cells contribute to vascular and mesenchymal lineages in zebrafish regeneration [29] and to CMs, smooth muscle, and endothelial cells during mouse development [30]. The epicardium further regulates CM proliferation via FGF- and IGF2-mediated signaling [31, 32]. Moreover, cytokine pathways such as IL11a-Stat3 signaling have been shown to activate epicardial fibroblast proliferation even in uninjured zebrafish hearts [33], highlighting the possibility that Stat3-mediated signals extend beyond CMs. Beyond the epicardium, Stat3 plays a central role in fibroblast biology, driving profibrotic gene expression and fibroblast-to-myofibroblast transition in part through crosstalk with TGF-β signaling [34, 35]. Thus, systemic or embryo-wide Stat3 activation—as in mRNA overexpression models—likely influences multiple non-myocyte compartments via paracrine cues, ECM remodeling, or cytokine secretion. Although our myocardium-specific TetON model mitigates this confounding, residual paracrine contributions from non-CMs remain possible.

In zebrafish, Jak/Stat3 signaling is robustly induced after cardiac injury, driving CM proliferation and regeneration [6]. Additional studies underscore its relevance: Il11/Stat3 signaling is indispensable for zebrafish cardiac regeneration [36], Il11 administration protects against myocardial infarction-induced apoptosis and fibrosis in mice [37], and Stat3 reactivation promotes CM cell cycle re-entry during myocarditis recovery [8]. Conversely, Stat3 deficiency impairs regeneration and exacerbates fibrosis [38]. Our findings extend these regenerative insights by showing that CM-specific Smarce1 deletion or Stat3 induction is sufficient to enhance CM proliferation even in the uninjured adult zebrafish heart. This highlights that epigenetic de-repression of Stat3 not only mimics injury-induced responses but can unlock proliferative programs under basal conditions, suggesting an intrinsic brake on Stat3 activity in the quiescent adult myocardium. These results raise important translational questions: can targeted epigenetic modulation of Stat3 bypass the postnatal mitotic block of mammalian CMs and thereby augment their limited regenerative capacity. Addressing this will be crucial for evaluating whether Smarce1-Stat3 signaling constitutes a viable entry point for therapeutic cardiac regeneration.

In summary, our study identifies Smarce1 as a pivotal chromatin regulator of Stat3 signaling, coordinating CM cell cycle activity and proliferation in both embryonic and adult zebrafish hearts. By bridging chromatin remodeling with pro-regenerative cytokine pathways, Smarce1 emerges as a candidate regulator whose modulation may inform strategies to promote heart regeneration in zebrafish and potentially in mammals.

## Experimental model and subject details

### Animals

All procedures and experiments in this study were carried out after appropriate institutional approvals (Tierforschungszentrum (TFZ) Ulm University; No. 0183, 24.03.2011; Regierungspräsidium Tübingen; No. 1415) and national (Germany) ethical and animal welfare regulation (Tierschutzgesetz § 11). The experimental procedures in this study were performed according to the guidelines from the EU Directive 2010/63/EU on the protection of animals used for scientific purposes. Care and breeding of zebrafish Danio rerio were carried out as described [39]. For injection experiments the TüAB wild-type strain was used. *Heart of stone* (*hos*) adult mutants were kept as heterozygous fish; breeding resulted in 25% homozygous *hos* mutant offspring. Bright-field images or videos of embryos were taken at 72- or 96 h post fertilization (hpf). For documentation, zebrafish embryos were treated with 0.003% 1-phenyl-2-thiourea to inhibit pigmentation. For immunofluorescence (IF) or heart-specific analyses, the Tg(*myl7*:mcherry.nls), Tg(*myl7*:GFP), Tg(*minUnc45b*:EGFP.CAAX), or Tg(*myl7*:mcherry-CAAX) lines were used, marking cardiomyocytes (CMs) with mcherry or GFP expression. For adult zebrafish experiment, 1-year-old of Tg(*myl7*:TetON-*smarce1*/AcGFP), Tg(*myl7*:TetON-*stat3*/AcGFP) zebrafish, or *smarce1*^fl/fl^ × Tg(*cryaa*:DsRed,-5.1*myl7*:CreERT2)^pd10^ were used.

### Generation of *smarce1* or *stat3*-inducible zebrafish models

To establish Tg(*myl7*:TetON-*smarce1*/AcGFP) or Tg(*myl7*:TetON-*stat3*/AcGFP), the vectors including zebrafish *smarce1* or *stat3*, Aequorea coerulescens-derived green fluorescence protein (AcGFP), the trans-activator protein as well as a modified tetracycline response element (TRE) cDNA were integrated into a destination vector for the zebrafish (pDestCS2 +) through the process of Gateway Cloning. The final plasmid was injected into the zebrafish embryo at 1-cell stage, and the zebrafish has been maintained to be homozygote. For creation of *smarce1*^fl/fl^, two LoxP sequences flanking *smarce1* were integrated by CRISPR/Cas9 to eliminate the *smarce1* with expression of the Cre recombinase. All injection and screening procedures were conducted by InVivo Biosystems.

## Method details

### EdU incorporation and immunofluorescence (IF) staining (embryonic hearts)

To capture distinct stages of the cell cycle across experimental contexts, we used complementary proliferation assays. Phospho-Histone H3 (pH3) immunostaining was used to quantify mitotic (M-phase) cardiomyocytes (CMs), EdU incorporation to quantify S-phase entry within a defined labeling window, and proliferating cell nuclear antigen (PCNA) immunostaining as a broader indicator of cycling/proliferative activity in embryonic hearts. For EdU incorporation assays in zebrafish embryo hearts, the Click-iT™ EdU Alexa Fluor™ 488 or 647 Imaging Kit was used. Embryos at 72- or 96 hpf were pulsed for 2 h with EdU on ice and fixed with 4% paraformaldehyde (PFA) in PBS for overnight at 4 °C. After fixation, hearts were dissected by use of 30G syringe needle and permeabilized in 1% Triton-X100 PBS before staining with EdU reaction cocktail. To perform pH3 staining, embryos were prepared at corresponding time points and fixed in 4% PFA overnight at 4 °C. Afterwards, samples were permeabilized in 2.5 mg/ml trypsin in 0.1% Triton in PBS (PBT) for 5 min on ice and blocked in 5% normal goat serum (NGS) in PBT for 1 h at room temperature (RT). Anti-PCNA or -pH3 (Ser10) (1:100) primary antibody was attached overnight at 4 °C, followed by corresponding Alexa-488 or -647 labelled secondary antibody incubation. All samples were mounted with VECTASHIELD® HardSet™ (with DAPI) for confocal imaging.

### RNA extraction and quantitative real-time PCR

Per biological replicate, 100 embryonic hearts collected at 96 hpf were pooled. RNA extraction was carried out by RNeasy Mini Kit according to the manufacturer's instructions. Total RNA (200 ng) was reverse transcribed to produce cDNA using Superscript III reverse transcriptase. Quantitative real-time PCR was performed according to standard protocols using SYBR Green on a LightCycler 480 II. Two house-keeping genes, *β-actin* and *18 s ribosomal RNA*, were used as reference gene for normalization of gene expression (List of primer sequences; Supplementary Table S2).

### Protein lysate extraction and Western blot analysis

For each of independent Western blot experiments, 50 embryos or dissected 100 embryonic hearts were used. Whole embryos were dechorionated manually and yolk was removed with deyolking buffer (55 mM NaCl, 1.8 mM KCl, 1.25 mM NaHCO_3_). Deyolked embryos were washed with washing buffer (110 mM NaCl, 3.5 mM KCl, 2.7 mM CaCl2, 10 mM Tris/HCl pH 8.5) for 3–4 times. Embryos were resuspended in RIPA buffer (10 mM Tris, 150 mM NaCl, 0.5 mM EDTA, 0.1% SDS, 1% TritonX-100, 1% sodium deoxycholate) containing protease inhibitor (cOmplete™) and phosphatase inhibitor (PhosSTOP™) then frozen in the liquid nitrogen for manual homogenization with pestles. Homogenized embryos were incubated on ice for 30 min and centrifuged for 15 min, at 18,000 g and 4 °C. Supernatant was collected and measured by Bradford assay. For Western blot analysis protein lysates were boiled in 5 × (protein lysate of whole mount embryos) or 2 × (embryonic heart tissues) Laemmli Buffer and loaded on a precast 8–16% SDS gel. Proteins were separated by SDS-PAGE and transferred to polyvinylidene fluoride (PVDF) membrane. After blocking in 5% skim milk powder in TBST (TBS with 0.1% Triton) for 2 h at RT, the membrane was incubated with the primary antibodies overnight at 4 °C. The primary antibodies used are listed in the Key resources table. Phospho-Stat3 was detected using an antibody recognizing zebrafish Stat3 Tyr708 (pStat3^Tyr708^), and we report phosphorylation site nomenclature accordingly throughout the manuscript. The corresponding anti-rabbit or -mouse IgG HRP-linked secondary antibodies were incubated for 2 h at RT after washing with TBST. Signals were detected by chemiluminescence, using a luminescent image analyzer (ImageQuant Las4000 mini). Western Blots were quantified by using ImageQuant LAS4000 software and normalized to ß-Actin of each protein targets and wt siblings.

### Imaging and counting CMs (embryonic hearts)

Whole mount zebrafish embryo images were taken by the Olympus SZX 16 microscope after orientation in 2.5% of methyl cellulose. Fluorescent images of the fluorescent CMs were taken with the Leica DMi8 confocal microscope with a 40 × objective (with oil). The total number of CMs in the entire ventricle or atrium was quantified. Hearts were dissected and high-resolution confocal z-stacks acquired at 0.44 µm intervals; every optical stack was then used for cell counting. For the confocal projections shown in the Figures, the full z-stacks were merged. This ensures that rare pH3- or EdU-positive CMs—often missed in individual stacks—are accurately visualized and represented. Counting of CM numbers was performed with the ImageJ cell counter plugin (particle analysis; point picker) and recounted for verification with Imaris (Oxford Instruments). Embryos were initially classified based on gross cardiac phenotype at 96 hpf, and genotypes were subsequently confirmed by PCR-based genotyping. For embryonic heart image-based quantifications (CM number and CM size), images were anonymized and quantified blinded to genotype/condition; group identities were revealed only after quantification was completed. Because embryos were collected based on phenotype for downstream analyses, randomization at the collection stage was not applicable; however, image acquisition and quantification were performed using identical settings and a predefined analysis workflow across groups. For statistics, the data was analyzed and visualized by using GraphPad Prism9.

### ATAC sequencing procedure and data analysis

For ATAC sequencing, 60 hearts of *hos* mutants and wt siblings were dissected from *hos*/Tg(*myl7*:GFP), flash frozen and sent to Active Motif for ATACseq library preparation and sequencing. Adapters and low‑quality bases were removed, mitochondrial/alignment artifacts filtered, and duplicates marked. Peaks were called per sample and merged across conditions to a consensus peak set. Peak accessibility was quantified as Tn5 insert pileups per consensus peak and analyzed with a quasi‑likelihood negative binomial model. Differentially accessible regions (DARs) were defined at FDR < 0.05 with an absolute logFC threshold; for inactivation‑focused analyses we emphasized decreased accessibility using logFC ≤  − 1. Peaks were annotated relative to the nearest transcription start site (TSS). Promoters were defined as TSS ± 2 kb; distal elements were peaks outside this window. When needed, gene-peak links were assigned by nearest TSS and, for distal peaks, constrained to the same topologically associated domain if available. GRCz11 (NCBI) was used as reference for further analyses. For differential analysis (e.g. heatmap), maxtag ((average value × length of region)/224, cutoffs: 100–200) and the position were considered for comparison. Average value of the tags per sample were compared and visualized using R. Chromatin regions that are differentially open in specific regions of interest were visualized and identified using Integrative Genomic Viewer.

### RNA sequencing procedures and data analysis

For heart-specific RNA-seq, three independent RNA samples for *hos* mutants and siblings (5 hearts/sample), were isolated and the RNA was extracted using the NucleoSpin RNA XS Kit according to manufacturer’s instructions. The libraries for RNA-seq were generated and sequenced on the Illumina HiSeq 2000 by the Genomics Core Facility at University Ulm (Single-end, 50 bp, 10 million reads per sample).

Processing of raw FASTQ files and alignment to Ensembl GRCz10 was done by the Genomics Core Facility at University Ulm. After creating the count matrix for Ensembl IDs and samples, low count reads (< ten counts per million in less than 3 samples) were removed. Counts were TMM normalized using the R/Bioconductor package edgeR [40]. The count data were further processed using the voom transformation, lmfit and eBayes functions included in the R/Bioconductor package limma [41]. The calculated DEGs were annotated using the R/Bioconductor package AnnotationDbi [42]. For comparison of wt and *hos* samples the mean value of the counts in each group were color coded for selected genes in a heatmap. The number of up- and down-regulated DEGs were counted, filtering for fold change (|log2(fold change)|> 1) and false discovery rate (FDR) < 0.05 and displayed in a bar plot. All identified DEGs were shown in a volcano plot by plotting the log2 of the fold change against the -log10 of the FDR highlighting the significantly deregulated genes in red or blue (|log2(FC)|> 1, FDR < 0.05). To test for over-representation of gene ontology (GO) terms the goana function of the limma package was used. Selected GO terms were shown in a barplot according to their -log10 (p-value).

### DEG-DAR integration and Motif enrichment with AME

We intersected DARs with the set of DEGs. For promoter analyses, only DARs overlapping TSS ± 2 kb of DEGs were retained. This yielded the integrative peak list used for motif discovery.

Motif enrichment was performed with AME (MEME Suite v5.5.8) on foreground FASTA sequences against curated vertebrate TF motif libraries. The primary database was JASPAR 2024 CORE vertebrates, redundant (v2); auxiliary analyses optionally included Jolma 2013 and UniPROBE mouse motif sets to assess robustness. Enrichment scoring was based on the total number of motif hits per sequence exceeding an internally determined threshold (“total-hits” method). Statistical significance was evaluated using Fisher’s exact test, comparing motif hit frequencies in the foreground sequences to those in a shuffled background. The hit threshold was optimized by AME’s internal true-positive (TP) threshold estimation (-hit-lo-fraction 0.25), which selects a position weight matrix (PWM) score cutoff that maximizes discriminatory power while minimizing low-score hits. Multiple testing correction was performed using the Benjamini–Hochberg false discovery rate (FDR) method, and motifs with an adjusted P-value (adj. P) < 0.05 were considered significantly enriched. For each motif, we also report the nominal P-value, E-value, TP (%) and FP (%)—representing the fraction of foreground and background sequences, respectively, containing at least one above-threshold motif occurrence—to facilitate interpretation.

### Single cell RNA sequencing and data analysis (embryonic hearts)

For heart dissection, wild-type (wt) and *smarce1*-deficient (*hos*) embryos were collected and anesthetized with tricaine at 96 hpf. Using a 1 mL syringe fitted with a 30G 1/2″ (13 mm) needle, 150–200 embryonic hearts per genotype were manually dissected and pooled, and each genotype pool was processed as a single independent scRNA-seq run. Dissected hearts were collected in a 1.5 mL tube containing cold DMEM and centrifuged at 700 rcf, 4 °C for 5 min. The supernatant was removed; samples were washed once with cold HBSS and centrifuged again under the same conditions. After removing the supernatant, pooled hearts were incubated with a mixed enzyme solution (100 μL papain + 5 μL thermolysin) in a Thermomixer at 300 rpm, 37 °C for 40 min to dissociate cells. The reaction was quenched with supplemented DMEM (+ 10% FBS), followed by centrifugation at 700 rcf, 4 °C for 5 min. After aspiration, dissociated cells were resuspended in PBS (+ 0.04% BSA) and counted. For single-cell mRNA capture, cDNA amplification, and library preparation, we used the SCOPE-chip (SD) RNA library kit according to the manufacturer’s protocol (Singleron). Dissociated cells were concentrated to 2.0–3.0 × 10^5^ cells/mL and loaded onto the microwell chip to capture 7,000–9,000 cells per run. Sequencing matrices were processed and analyzed using CeleLens Cloud (Singleron) with a Scanpy-based backend (scanpy v1.9.3; anndata v0.7.8). For quality control, we applied the platform filtering parameters corresponding to Singleron-recommended settings: minimum detected genes = 200 (mingene = 200), upper quantile cutoffs for detected genes and UMIs (maxgene = 0.98; maxUMI = 0.98), and mitochondrial filtering using a predefined threshold (5, 10, 15, 20, 30, or 50) selected to approximate removal of ~ 5% of cells, with ribosomal content filtering set to < 50%. After parsing and QC, the dataset contained 2,148 cells for the wt sibling control and 4,025 cells for the *hos* mutant sample. For this analysis, we did not apply ambient RNA correction (decontX), doublet detection/removal (Scrublet), cell-cycle regression, batch-effect correction, or sample integration. To normalize gene expression for downstream analyses, ribosomal protein genes (RPL and RPS) were excluded and standard single-cell preprocessing practices consistent with commonly used Seurat workflows were followed [43]. Cells were annotated using referenced cell type markers [44] and visualized by UMAP. Clustering was performed at resolution 1.2 using 2,000 highly variable genes and a Singleron-recommended number of principal components. Differential expression analyses and gene expression visualization were performed within CeleLens Cloud. CMs were analyzed at the level of major cell-type annotations, without atrial/ventricular or cell-cycle phase-resolved subclustering.

### Microinjection

Microinjections were performed into fertilized zebrafish oocytes at the 1-cell stage, using pulled glass capillaries and a Microinjector. Embryos were then allowed to develop at 28.5 °C until the indicated stages. Morpholino-modified antisense oligonucleotides (MOs; Gene Tools, LLC) were injected into one-cell stage zebrafish embryos. To knockdown zebrafish Stat3, MO targeting the translational start site (*stat3* MO: 5´-GCCATGTTGACCCCTTAATGTGTCG-3´) or 5-base-pair mismatch MO as control (*stat3* mmMO: 5´-GCCtTGTaGACCCcTTAAaGTGaCG-3’) were injected into fertilized oocytes at the one-cell stage. MOs were injected with 2.5 ng in 0.2 M potassium chloride. For *stat3* overexpression, zebrafish *stat3* cDNA was amplified by Q5® High-Fidelity DNA Polymerase and cloned into donor plasmid (pDONR221) and destination vector (pDestCS2 +) by Gateway Cloning system. Capped RNA of zebrafish *stat3* was synthesized from *stat3* cDNA inserted pCS2 + vector using the mMESSAGE mMASCHINE system and 0.25 ng of mRNA in 0.2 M potassium chloride were injected.

### Pharmacological inhibition of Stat3 activity

To inhibit Stat3 activation, Stat3 phosphorylation inhibitor, Stattic (22), was used. By screening different concentration of Stattic (1–20 µM) with different incubation time (0–96 h) in zebrafish embryos, an optimal concentration (5 µM) of Stattic and incubation time (0–96 hpf) were determined, which reduces pStat3^Tyr708^ levels without phenotypes of developing zebrafish embryos at 96 hpf. The efficiency of Stattic was confirmed by Western Blot.

### Intraperitoneal injection into adult zebrafish (tamoxifen, doxycycline, and EdU)

Intraperitoneal injection was performed as shown in Figs. 5A and 6A. In the experiment of *smarce1* loss-of-function, *smarce1*^fl/fl^(*myl7*:CreERT2) zebrafish were injected with 10 µl of 4-hydroxytamoxifen (4-OHT; 1.25 mM) in PBS. To activate *smarce1*- or *stat3* TetON zebrafish, 10 µl of doxycycline (1.125 mM) in PBS was injected every 2 days after 3 days of consecutive injections. Proliferative capacity of CMs were evaluated by daily injection of 10 mM 5-ethynyl-2-deoxyuridine (EdU) in PBS for 3 days, starting 3 days before heart isolation.

### EdU incorporation assay in adult zebrafish tissue sections

Zebrafish hearts were fixed in 4% paraformaldehyde (PFA) (in phosphate buffer with 4% sucrose) at RT for 1 h, washed three times for 10 min in 4% sucrose/phosphate buffer and equilibrated in 30% sucrose/phosphate buffer overnight at 4 °C. Hearts were embedded and cut into 10 µm sections. Sections were equally distributed onto 8 serial slides so that each slide contained sections representing all areas of the ventricle. For EdU detection, EdU incorporated cryosections were thawed at RT for 1 h and washed three times with PEMTx (PEM (8 mM NA-PIPES, 0.5 mM EGTA, 1 mM MgCl_2_∙6H_2_O) + 0.2% Triton-X-100) for 10 min. After incubation with 50 mM NH_4_Cl in PEMTx for 10 min, the sections were washed twice with PEMTx for 10 min. Slides were washed twice in 3% BSA/PBS for 10 min and incubated in the dark with the EdU reaction cocktail by following manufacturer’s protocol for 1 h at RT. Slides were washed for 10 min in 3% BSA/PBS and covered with VectorShield (with DAPI). All fluorescent images of cryosections are stitched after tiles imaging by Leica TSC SP8 confocal microscopy. Quantifications of the fraction of EdU^+^ CMs were performed on 2 to 3 sections in the ventricles. For each heart the average value was calculated from the analysis of 2–3 sections per heart. Blinding was applied to embryonic IF-based quantifications only and was not applied to omics analyses or adult experiments.

### Statistical analysis

All graphs and statistical analyses are presented as mean ± standard deviation (SD). Statistical analyses were performed using GraphPad Prism 9. For comparisons between two groups, statistical significance was assessed using an unpaired two-tailed Student’s t-test; when data did not satisfy assumptions for parametric testing, the Mann–Whitney test was used as implemented in Prism. For comparisons among three or more groups, one-way ANOVA was applied as appropriate, followed by Holm-Sidak’s multiple-comparisons test. Normality and variance were assessed in Prism to guide the choice of parametric versus non-parametric tests. A p-value < 0.05 was considered statistically significant. Exact n values, what n represents (e.g., embryos, pooled hearts, independent clutches/biological replicates), and the unit of analysis are reported in the corresponding figure legends. Where cell-level measurements were performed (e.g., CM size), the number of cells analyzed is additionally indicated in the figure legends. Effect sizes are reported as mean differences with 95% confidence intervals (CIs) for primary image-based quantitative endpoints (e.g., CM numbers, numbers of marker-positive CMs, and derived indexes). For qRT-PCR and immunoblot densitometry, values are reported as normalized fold-changes (mean ± SD) with exact n and p-values; mean-difference CIs are not additionally reported because these readouts are inherently normalization- and scale-dependent.

## Supplementary Information

Below is the link to the electronic supplementary material.

Supplementary Material 1

## Data Availability

All supporting data are available within the article and its Supplemental Material. Detailed materials and methods can be found in the Supplemental Material. ATAC-seq and RNA-seq data from this study have been deposited in the Gene Expression Omnibus database under accession number GSE223722. Resource Availability: Further information and requests for resources and reagents should be directed to and will be fulfilled by the Lead Contact, Steffen Just (steffen.just@uniklinik-ulm.de). Material Availability: All unique/stable reagents generated in this study are available from the Lead Contact with a completed Materials Transfer Agreement (Key resources table; Supplementary Table S1).
