# Supplementary material for: Smarce1-dependent modulation of Stat3 signaling governs cardiomyocyte proliferation
Source: Biol Res. 2026 Mar 10;59:21. doi: 10.1186/s40659-026-00681-2 (PMC13064369; doi:10.1186/s40659-026-00681-2)
Supplement: Supplementary file 1 — Supplementary Material 1 [file 40659_2026_681_MOESM1_ESM.docx]

**Supplementary Table S1.** Key resources table

| **REAGENT or RESOURCE** | **SOURCE** | **IDENTIFIER** |
| --- | --- | --- |
| **Chemicals** | | |
| Proteinase K, recombinant, PCR Grade | Roche | Cat#3115844001 |
| 1-Phenyl-2-thiourea | Sigma-Aldrich | Cat#P7629 |
| Ethyl-3-aminobenzoate methanesulfonate salt | Sigma-Aldrich | Cat#A5040 |
| Paraformaldehyde (PFA) | Sigma-Aldrich | Cat#158127 |
| JB-4® Embedding Kit | Polyscience | Cat#00226-1 |
| Methyl Cellulose | Sigma-Aldrich | Cat#64632 |
| Trypsin (powder) | Biochrom | Cat#L2103 |
| VECTASHIELD® HardSet™ with DAPI | Vector Laboratories | Cat#H-1500 |
| Normal Goat Serum (NGS) | Sigma-Aldrich | Cat#S26-100ML |
| Triton^®^ X-100 | Serva Electrophoresis | Cat#37240 |
| Invitrogen^TM^ RNaseOUT™ Recombinant Ribonuclease Inhibitor | Thermo Fisher Scientific | Cat#10777-019 |
| Light Cycler 480 SYBR Green I Master Mix | Roche | Cyt#04887352001 |
| Nuclease free water | QIAGEN | Cat#129114 |
| cOmplete™, EDTA-free Protease Inhibitor | Roche | Cat#04693132001 |
| PhosSTOP™, Phosphatase Inhibitor | Roche | Cat#04906837001 |
| Protein Assay Dye Reagent Concentrate (Bradford) | Bio-Rad | Cat#5000006 |
| 8–16% Mini-PROTEAN® TGX™ Precast Protein Gels, 10-well | Bio-Rad | Cat#456-1104 |
| 10x Tris/Glycine/SDS | Bio-Rad | Cat# 1610732 |
| 10x Tris/Glycine Buffer | Bio-Rad | Cat# 1610734 |
| PageRuler™ Plus Prestained Protein Ladder | Thermo Fisher Scientific | Cat# 26619 |
| TWEEN 20 | Sigma-Aldrich | Cat#P1379 |
| Skim Milk Powder | Sigma-Aldrich | Cat#70166 |
| Amersham™ ECL Prime | Cytiva | Cat#RPN2232 |
| Immobilon-P PVDF-Membran | Millipore | Cat#IPVH00010 |
| Not I-HF | New England BioLabs | Cat#R3189L |
| Stattic | Selleckchem | Cat#S7024 |
| **Antibodies** | | |
| Cyclin A1 | Aviva System Biology | Cat#OAAJ-01741 |
| Cyclin B1 | MybioSource | Cat#MBS9205589 |
| Cyclin D1 (DCS6) | Cell Signaling | Cat#2926 |
| Cyclin E1 | Proteintech | Cat#11554-1-AP |
| PCNA | GeneTex | Cat#GTX124496 |
| p21_CDKN1A | Aviva System Biology | Cat#ARP30198_P050 |
| p27 Kip1 | GeneTex | Cat#GTX100446 |
| Anti-beta-actin | Sigma Aldrich | Cat#A5441 |
| Smarce1 | Aviva System Biology | Cat#ARP38224_P050 |
| IL-11RA | Proteintech | Cat#10264-1-AP |
| IL-6ST | Biorbyt | Cat#orb393081 |
| JAK1 (6G4) | Cell Signaling | Cat#3344 |
| STAT3 (124H6) | Cell Signaling | Cat#9139 |
| Phospho-Stat3 (Tyr708) | MBL Life Science | Cat#D128-3 |
| Phospho-STAT3 (Ser727) | Cell Signaling | Cat#9134 |
| Anti-phospho-Histone H3 (Ser10) | Millipore | Cat#06-570 |
| Anti-mouse IgG, HRP-linked | Cell Signaling | Cat#7076S |
| Anti-rabbit IgG, HRP-linked | Cell Signaling | Cat#7074S |
| Goat anti-Rabbit IgG (H+L) Highly Cross-Adsorbed Secondary Antibody, Alexa Fluor 647 | Thermo Fisher Scientific | Cat#A-21245 |
| **Bacterial Strain** | | |
| One Shot TOP10 Electrocomp E.coli | Thermo Fisher Scientific | Cat#C404050 |
| **Critical Commercial Assays** | | |
| NucleoSpin RNA Plus XS | Machery-Nagel | Cat#MN740990250 |
| Click-iT® EdU Alexa Fluor® 647 Imaging Kit | Thermo Fisher Scientific | Cat#C10340 |
| Morpholino | GENE TOOLS, LLC | https://www.gene-tools.com |
| TOPO TA Cloning Kit Dual Promotor | Thermo Fisher Scientific | Cat#45-0640 |
| RNeasy Micro Kit | QIAGEN | Cat#74004 |
| Superscript III Reverse Transcriptase | Thermo Fisher Scientific | Cat#18080-085 |
| Q5^®^ High-Fidelity DNA Polymerase | New England BioLabs | Cat#M0491L |
| Isolate II PCR and Gel Kit | Biocat | Cat#BIO-52060 |
| Gateway Cloning | Thermo Fisher Scientific | Cat#11789100  Cat#11791020 |
| mMESSAGE mMASCHINE SP6 | Ambion | Cat#AM1340 |
| ATAC-seq service | Active Motif | https://www.activemotif.com |
| TubeSeq service | Eurofins Genomics | https://eurofinsgenomics.eu/ |
| Next Generation Sequencing (RNA sequencing) | Genomics Core Facility at University Ulm | https://www.uni-ulm.de/medizinische-fakultaet/forschung/core-facilities/genomics/ |
| **Experimental Models: Animal** | | |
| Zebrafish | . | Tg(*myl7*:mcherry.*nls*) |
|  | . | Tg(*myl7*:GFP)) |
|  | Steffen Just lab (this paper) | Tg(*minUnc45b*:EGFP.CAAX) |
|  | . | Tg(*myl7*:mcherry.CAAX) |
|  | Steffen Just lab (this paper) | Smarce1^flox/flox^(*myl7*:CreERT2) |
|  |  | Smarce1 TetON(*myl7*:AcGFP.nls) |
|  |  | Stat3 TetON(*myl7*:AcGFP.nls) |
| **Oligonucleotides** | | |
| Oligo dTs | Eurofins | N/A |
| dNTP Mix | Thermo Fisher Scientific | Cat#R1122 |
| **Recombinant DNA** | | |
| pDonor221 | Thermo Fisher Scientific | Cat#12536017 |
| pCS2+ | Addgene | N/A |
| **Software and Algorithms** | | |
| R (version 3.5.1) | CRAN | https://www.r-project.org/ |
| edgeR | R/Bioconductor | https://rdocumentation.org/packages/edgeR/versions/3.14.0 |
| limma | R/Bioconductor | https://rdocumentation.org/packages/limma/versions/3.28.14 |
| AnnotationDbi | R/Bioconductor | https://rdocumentation.org/packages/AnnotationDbi/versions/1.34.4 |
| Integrative Genomic Viewer | Broad Institute  and the Regents of the  University of California | http://software.broadinstitute.org/software/igv |
| GraphPad Prism (version 9) | GraphPad Software, La Jolla California, USA | https://www.graphpad.com:443 |
| ImageJ | NIH | https://imagej.nih.gov/ij/ |
| Imaris | Oxford Instruments | https://imaris.oxinst.com/ |

**Supplementary Table S2.** List of primer sequences

| **Experiment** | **Gene** | **Forward sequence (5´-3´)** | **Reverse sequence (5´-3´)** |
| --- | --- | --- | --- |
| Genotype | *smarce1* | TTCTGAGTGAGGTGGTGGTG | AGAGCCGTTTGAGCTCGTTA |
| Geteway cloning | *zstat3* | GGGGACAAGTTTGTACAAAAAAGCAGGCTACATGGCGAGTCAAGTTGCAGT | GGGGACCACTTTGTACAAGAAAGCTGGGTACTAAGCATTTCGGCAGGTGTC |
| Real-time PCR | *cdkn1a* | GCTGCACTCCCGCATGAAGT | CACTAGACGCTTCTTGGCTTGGT |
|  | *cdkn1ba* | TCAGCACGCCGAGGAAACGA | CTGGCGAAGTAGTCGATGGTGAG |
|  | *cdkn1bb* | ACGGGAATCACGACTGTAGGGTAA | TCTGGGCGTTCGGGTCACTT |
|  | *cdkn1ca* | AGGCGATTTCAGAGGACACTTTGC | GGAAGCGTCTCCTGTTGCGTTAA |
|  | *cdkn1cb* | TCGACGATGTCTACCGTCCT | TTTCCTGGATGCCAGTCTTT |
|  | *cdkn2ab* | GTAACGCACCTTTGGCTCTT | CGGGGTCAGGCACATTAG |
|  | *ccna1* | CACTCTAAATGGGGCTTTGTG | CCCGAGATGGAGCTTATGAA |
|  | *ccnb1* | GAAGAAGGAGGTGAAGGTTGC | AGGAGAGGAAGGCTCAGACA |
|  | *ccne1* | GCAGCTCAGCCCTTAAGACA | GCGGATAGGAGTGAACCTTTC |
|  | *cdk1* | TTGGGGTCCCAGTAAGAGTCTA | GGTGTGGAATAGCGTGAAGC |
|  | *cdk2* | TGACATCTGGAGTTTGGGCT | AGGAGGGTTTGTAGTCTGGC |
|  | *cdk4* | AGGACATACCTGGAGAAAGTTCC | AGCTGCTGCATCAGGTCTC |
|  | *cdk6* | TGGCTCTTACTTCCGTGGTT | AGCTGGACTGAAGCAGCAC |
|  | *smarce1* | CCGAGCTGCTTCAAATCGAG | AGAGCCGTTTGAGCTCGTTA |
|  | *il11a* | CACCGGTTCAAGTCTCTTCC | CCTCTAGAGTTGGGAAGTCACTG |
|  | *il11ra* | ACATGGTGGAGGCCAAAC | TGTCCTCACTGGATAGGACTGA |
|  | *il6* | ACGCGAATCTACAGCGTCCT | CACCTGCAGCTGGCTGTTTA |
|  | *il6st* | GAGAATCATCCCGGCGAGAG | GCATCATCCACAACGGGAGA |
|  | *jak1* | AAACACATCGCCCTGCTCTA | AAAGGGCCGTACTGAACAAA |
|  | *socs3a* | TAAAGCAGGGAAGACAAGAGCCGA | TGGAGAAACAGTGAGAGAGCTGGT |
|  | *socs3b* | CGGATAACGCTTTGAAGCTGCCTT | TACTATGCGTTACCATGGCGCTCT |
|  | *stat3* | CCCATGGAGCTCCGACAGTT | ACGATGCGGGCAATCTCCAT |
|  | *ccnd1* | ACCTCACCAACTTCCTCTC | ACTCGATCTGTTCCTGACAC |
|  | *β-actin* | GCAGAAGGAGATCACATCCCTGGC | CATTGCCGTCACCTTCACCGTTC |
|  | *18s rRNA* | CACTTGTCCCTCTAAGAAGTTGCA | GGTTGATTCCGATAACGAACGA |


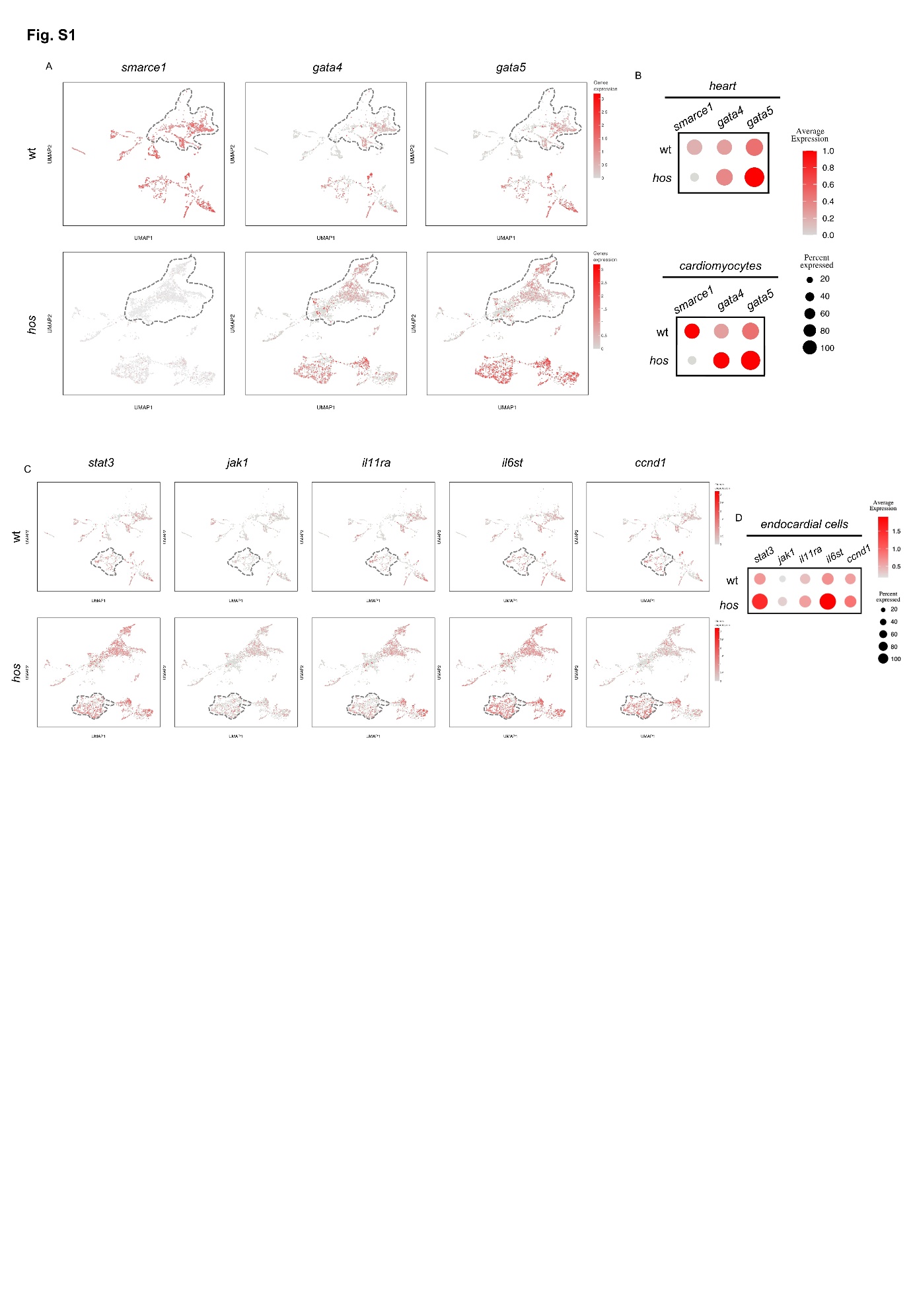


**Figure. S1: Spatial expression of *smarce1*, *gata4*, *gata5*, and Jak/Stat3 pathway genes across cardiac cell populations identified by single-cell RNA sequencing.**

(A) Feature plots showing the spatial expression patterns of *smarce1*, *gata4*, and *gata5* across all cardiac cell populations. Gene expression is indicated in red, with color intensity reflecting relative transcript abundance. Gray dashed lines delineate the cardiomyocyte cluster. (B) Dot plots illustrating the relative expression levels and the proportion of expressing cells for *smarce1*, *gata4*, and *gata5*. *Smarce1* expression is markedly reduced, whereas *gata4* and *gata5* are upregulated in both whole-heart and cardiomyocyte populations of *hos* embryos compared to wt. (C) Gene expression feature plots illustrating the spatial expression patterns of *stat3*, *jak1*, *il11ra*, *il6st*, and *ccnd1* (colored in red). Color intensity represents relative transcript abundance. Gray dashed lines delineate the endocardial cell cluster. (D) Dot plots showing the relative expression levels and the proportion of expressing cells for *stat3*, *jak1*, *il11ra*, *il6st*, and *ccnd1* in endocardial cell population.

**
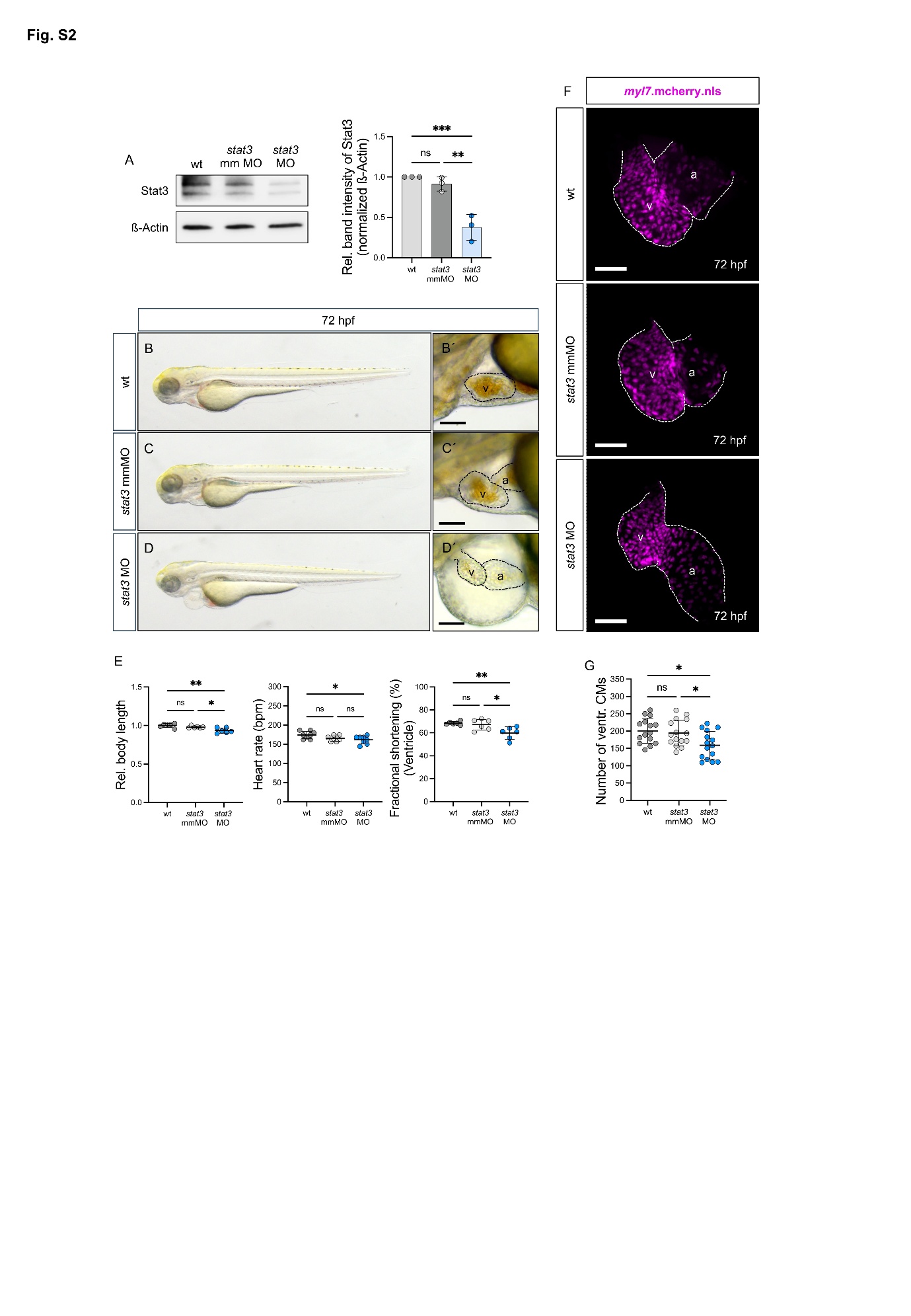
**

**Figure. S2: Knockdown of Stat3 causes cardiac defects in the developing zebrafish heart.**

(A) Western Blot bands showing protein expression of Stat3 and ß-Actin in KCl-, *stat3* mmMO-, and *stat3* MO-injected embryos at 72 hpf. Quantification of Western Blot band intensity showing knockdown of Stat3 by *stat3* MO injection in zebrafish embryos at 72 hpf (*stat3* mmMO: 0.92 ± 0.09, *stat3* MO: 0.38 ± 0.16, mean difference (*stat3* MO - *stat3* mmMO): -0.54, 95% CI: -0.87 to -0.21, SD, n = 3 (independent biological replicates; pooled-heart lysates), ns: p > 0.05, **p < 0.01, ***p < 0.001). (B-D) Lateral view of wt embryos injected with KCl, *stat3* mismatch (mm)- or *stat3*-morpholino (MO) at 72 hpf. (B´-D´) Magnified images of wt and MOs-injected embryos (scale bar: 50 µm). *Stat3* MO-injected embryo showed a cardiac edema and short body length. (E) Quantitative analyses of relative body length, heart rate, and ventricular fractional shortening of KCl-, *stat3* mmMO-, and *stat3* MO-injected embryos at 72 hpf (Rel. body length: wt: 1.00 ± 0.03, *stat3* mmMO: 0.98 ± 0.01, *stat3* MO: 0.94 ± 0.03, SD, n = 6 (embryos); Heart rate: wt: 174.00 ± 9.62, *stat3* mmMO: 165.00 ± 7.17, *stat3* MO: 161.25 ± 10.36, SD, n = 8 (embryos); Fractional shortening: wt: 68.41 ± 1.56, *stat3* mmMO: 67.10 ± 4.68, *stat3* MO: 59.72 ± 5.40, SD, n = 6 (embryos), ns: p > 0.05, *p < 0.05, **p < 0.01). (F) Confocal microscopy images of fluorescent cardiomyocyte (CM) nuclei (*myl7*.nls) in the hearts of KCl-, *stat3* mmMO-, and *stat3* MO-injected embryos at 72 hpf (scale bar: 50 µm). (G) Quantification of CM numbers in KCl-, *stat3* mmMO-, and *stat3* MO-injected embryonic hearts at 72 hpf (wt: 200.87 ± 36.29, *stat3* mmMO: 194.47 ± 37.44, *stat3* MO: 159.07 ± 40.30, SD, n = 15 (embryonic hearts), ns: p > 0.05, *p < 0.05). v = ventricle, a = atrium, ventr. = ventricular, Rel. = Relative.


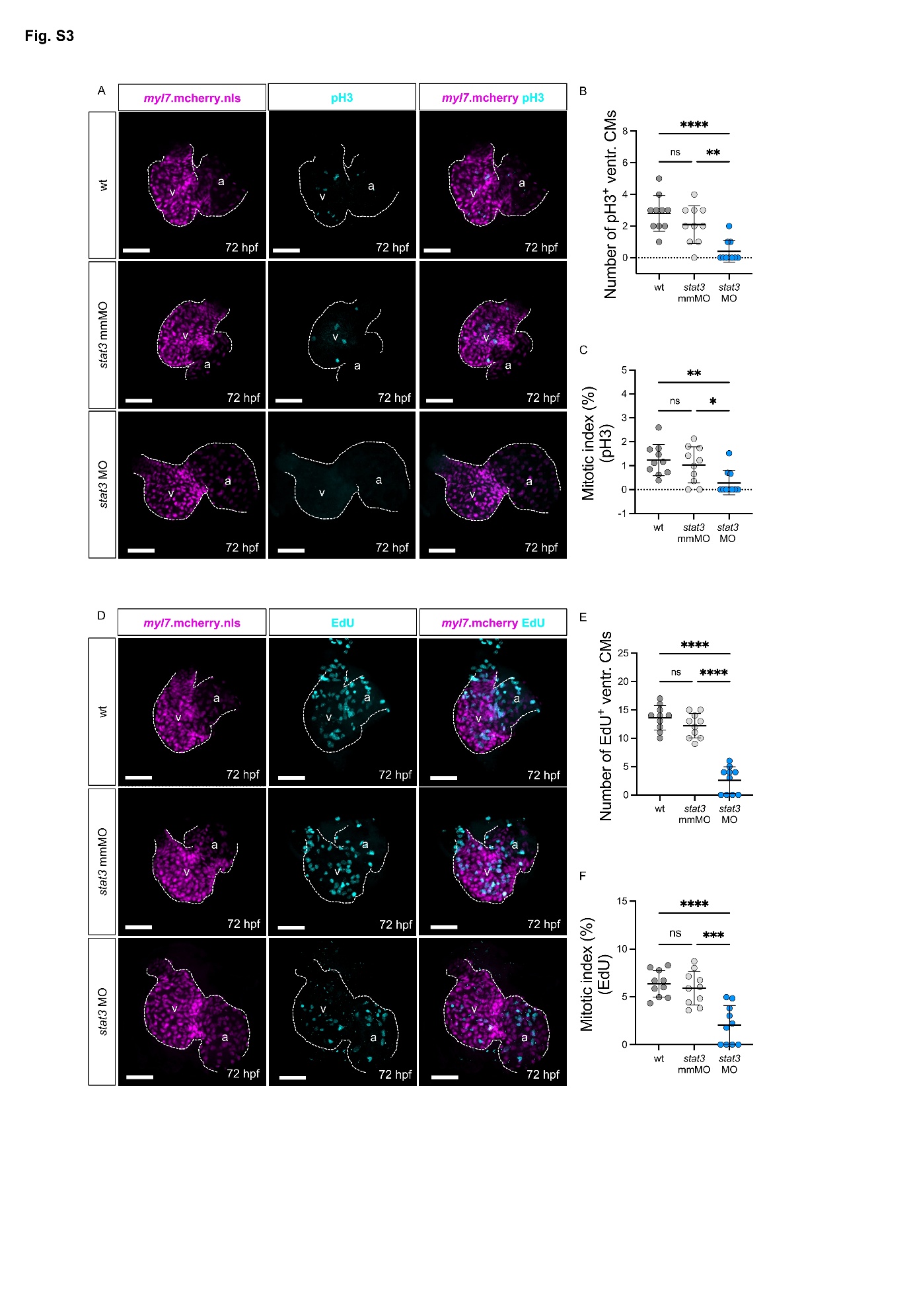


**Figure. S3: MO-mediated Stat3 knockdown reduces CM proliferation in zebrafish embryonic hearts.**

(A) Immunofluorescence (IF) staining of phosphorylated histone 3 (pH3) visualizing M-phase of cell proliferation in embryonic zebrafish hearts of KCl-, *stat3* mmMO-, and *stat3* MO-injected embryos at 72 hpf (scale bar: 50 µm). (B-C) Quantification of pH3-positive (pH3^+^) CMs and mitotic index (pH3^+^ CMs/total CMs) in the hearts of KCl-, *stat3* mmMO-, and *stat3* MO-injected embryos at 72 hpf (Number of pH3^+^ ventr. CMs: wt: 2.80 ± 1.14, *stat3* mmMO: 2.10 ± 1.20, *stat3* MO: 0.4 ± 0.70; Mitotic index: wt: 1.23 ± 0.65, *stat3* mmMO: 1.03 ± 0.76, *stat3* MO: 0.29 ± 0.52, SD, n = 10 (embryonic hearts), ns: p > 0.05, *p < 0.05, **p < 0.01, ****p < 0.0001). (D) Dissected hearts of Tg(*myl7*:mcherry.nls) incorporated with EdU visualizing DNA synthesis indicating S phase of cell cycle at 72 hpf (scale bar: 50 µm). (E-F) Quantification of EdU-positive (EdU^+^) CMs and mitotic index in the hearts of KCl-, *stat3* mmMO-, and *stat3* MO-injected embryos at 72 hpf (Number of EdU^+^ ventr. CMs: wt: 13.60 ± 2.17, *stat3* mmMO: 12.20 ± 2.15, *stat3* MO: 2.6 ± 2.37; Mitotic index: wt: 6.36 ± 1.40, *stat3* mmMO: 5.91 ± 1.76, *stat3* MO: 2.06 ± 2.03, SD, n = 10 (embryonic hearts), ns: p > 0.05, ***p < 0.001, ****p < 0.0001). Stat3 knockdown reduces CM proliferative potential. v = ventricle, a = atrium, ventr. = ventricular.


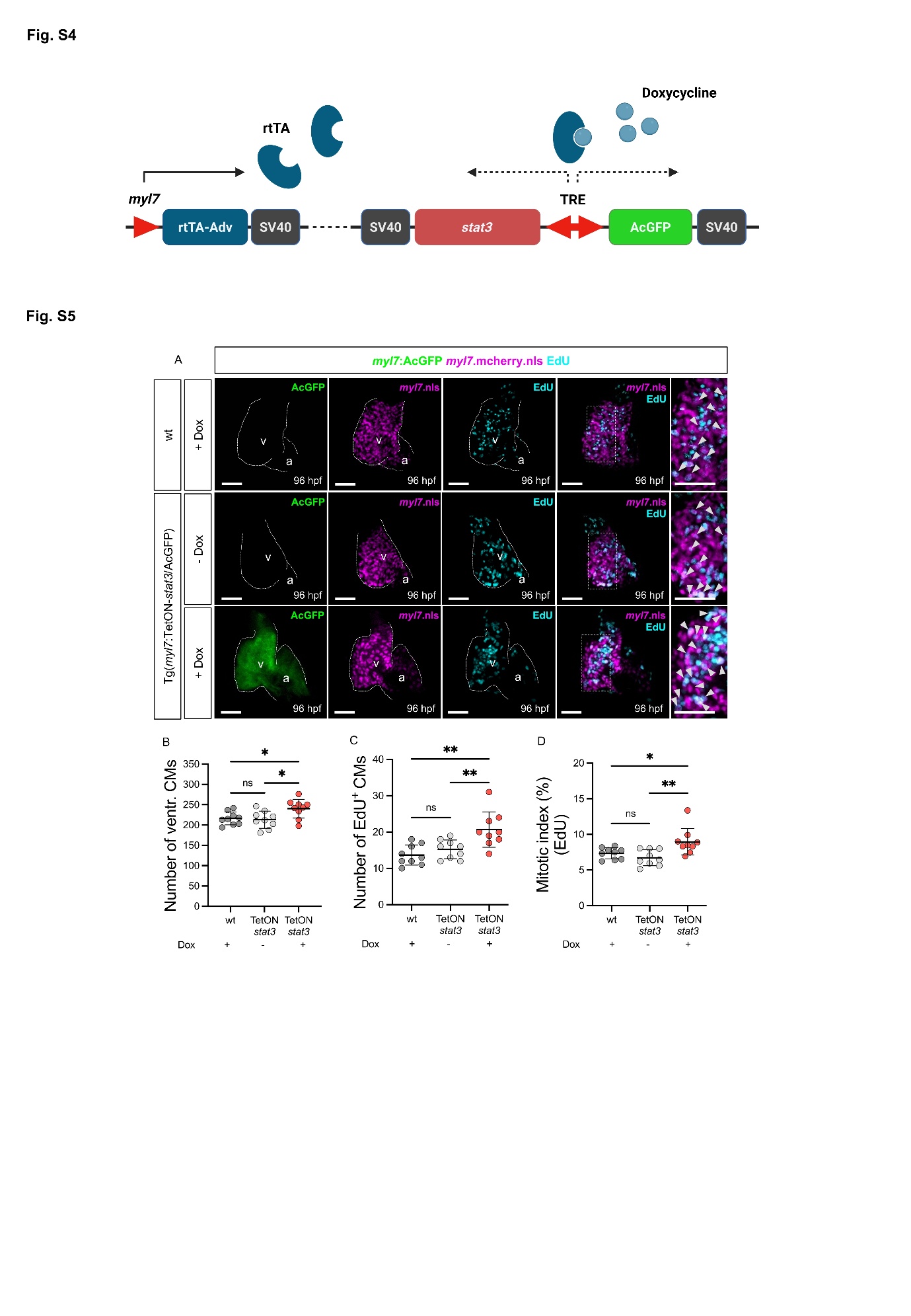


**Figure. S4:** **Construction of doxycycline-dependent TetON system inducing myocardial *stat3* overexpression.**

(A) Illustration of *myl7*:TetON-*stat3*/AcGFP construct. The protein of rtTA-Adv is specifically expressed in myocardium by promoter of *myl7*. Under doxycycline treatment, *stat3* and AcGFP are bidirectionally induced in myocardium (Biorender.com).


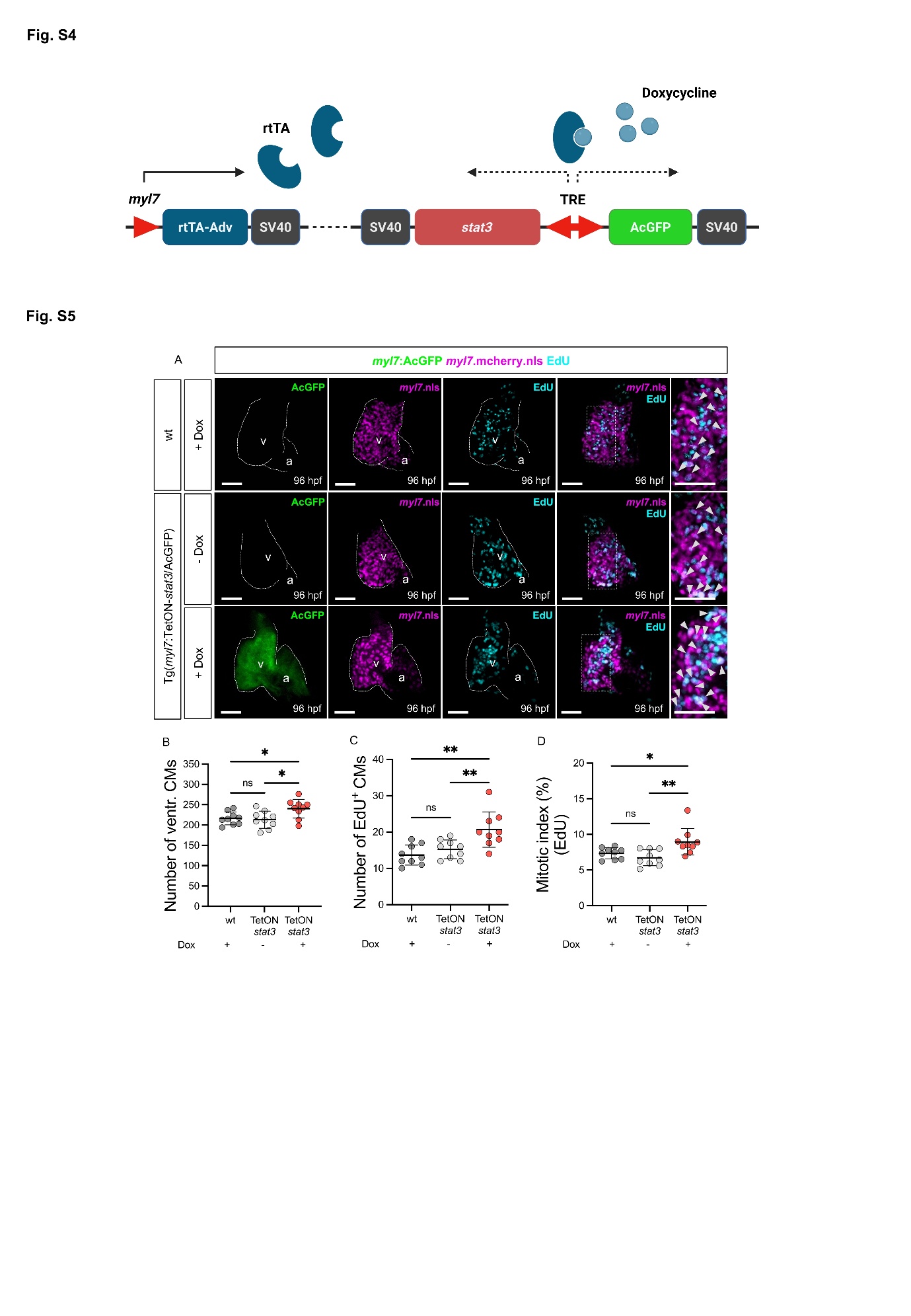


**Figure. S5: Myocardial *stat3* overexpression enhances CM proliferation in the developing zebrafish heart.**

(A) Confocal projections of Tg(*myl7*:mcherry.nls) or Tg(*myl7*:TetON-*stat3*/AcGFP) × Tg(*myl*7:mcherry.nls) hearts incorporated with EdU at 96 hpf (scale bar: 50 µm). (B-D) Quantitative analyses of ventricular CMs, EdU^+^ ventricular CMs, and mitotic index in wt or Tg(*myl7*:TetON-*stat3*/AcGFP) hearts at 96 hpf (Number of ventr. CMs: wt + dox: 216.22 ± 16.04, TetON-*stat3* - dox: 213.22 ± 20.99, TetON-*stat3* + dox: 240.22 ± 23.14; Number of EdU^+^ CMs: wt + dox: 13.67 ± 2.78, TetON-*stat3* - dox: 15.22 ± 2.59, TetON-*stat3* + dox: 20.67 ± 4.90; Mitotic index: wt + dox: 7.34 ± 0.79, TetON-*stat3* - dox: 6.72 ± 1.11, TetON-*stat3* + dox: 8.96 ± 1.86, SD, n = 9 (embryonic hearts), ns: p > 0.05, *p < 0.05, **p < 0.01). v = ventricle, a = atrium, ventr. = ventricular.
